# Citrate Accumulation-Related Gene Expression and/or Enzyme Activity Analysis Combined With Metabolomics Provide a Novel Insight for an Orange Mutant

**DOI:** 10.1038/srep29343

**Published:** 2016-07-07

**Authors:** Ling-Xia Guo, Cai-Yun Shi, Xiao Liu, Dong-Yuan Ning, Long-Fei Jing, Huan Yang, Yong-Zhong Liu

**Affiliations:** 1 Key Laboratory of Horticultural Plant Biology (Huazhong Agricultural University), Ministry of Education, Wuhan 430070, P.R. China; 2 College of Horticulture & Forestry Sciences, Huazhong Agricultural University, Wuhan 430070, P.R. China

## Abstract

‘Hong Anliu’ (HAL, *Citrus sinensis* cv. Hong Anliu) is a bud mutant of ‘Anliu’ (AL), characterized by a comprehensive metabolite alteration, such as lower accumulation of citrate, high accumulation of lycopene and soluble sugars in fruit juice sacs. Due to carboxylic acid metabolism connects other metabolite biosynthesis and/or catabolism networks, we therefore focused analyzing citrate accumulation-related gene expression profiles and/or enzyme activities, along with metabolic fingerprinting between ‘HAL’ and ‘AL’. Compared with ‘AL’, the transcript levels of citrate biosynthesis- and utilization-related genes and/or the activities of their respective enzymes such as citrate synthase, cytosol aconitase and ATP-citrate lyase were significantly higher in ‘HAL’. Nevertheless, the mitochondrial aconitase activity, the gene transcript levels of proton pumps, including vacuolar H^+^-ATPase, vacuolar H^+^-PPase, and the juice sac-predominant p-type proton pump gene (*CsPH8*) were significantly lower in ‘HAL’. These results implied that ‘HAL’ has higher abilities for citrate biosynthesis and utilization, but lower ability for the citrate uptake into vacuole compared with ‘AL’. Combined with the metabolites-analyzing results, a model was then established and suggested that the reduction in proton pump activity is the key factor for the low citrate accumulation and the comprehensive metabolite alterations as well in ‘HAL’.

An orange mutant named ‘Hong Anliu’ (HAL, *Citrus sinensis* cv. Hong Anliu) was first characterized by its high accumulation of lycopene, lower acid and higher soluble sugars in the fruit juice sacs during the ripening stage^1^. A recent metabolic analysis by Pan *et al*.^2^ indicated that many secondary metabolites, such as flavonoids, amino acids and lipids, also showed significant differences compared with the wild type orange ‘Anliu’ (AL). In the past few years, many researches have been done to investigate the possible reason for high accumulation of lycopene in ‘HAL’^1^,^3^,^4^,^5^,^6^. Although precious researches also identified many differentially expressed genes or differential proteins in ‘HAL’ as compared with in ‘AL’^4^,^5^,^6^, a possible mechanism to explain the comprehensive metabolite change in the mutant is not available at present.

In citrus fruit juice sacs, soluble carbohydrates, carotenoids, and some specific secondary metabolites accumulate and organic acid reduces in general as the fruit ripens^7^,^8^,^9^. The carbohydrates in the fruits are primarily from the source leaves, whereas the organic acids and other secondary metabolites synthesize locally in the fruits^8^,^10^. The carbon skeletons for all the locally synthesized metabolites come from carbohydrate catabolism through glycolysis and the tricarboxylic acid (TCA) cycle. Glycolysis is the metabolic pathway that converts glucose into pyruvate, which transports actively into the mitochondrion where it oxidizes to produce acetyl-CoA or forms oxaloacetate (OAA) by the carboxylation. Moreover, phosphoenolpyruvate (PEP), which is an intermediate in glycolysis, can also form OAA by the catalysis of phosphoenolpyruvate carboxylase (PEPC, EC4.1.1.31). The TCA cycle begins with the condensation of acetyl-CoA and OAA to form citrate catalyzed by citrate synthase (CS, EC2.3.3.1). Subsequently, aconitase (Aco, EC4.2.1.3) isomerizes the citrate to isocitrate. Then, isocitrate dehydrogenase (IDH, EC1.1.1.42) dehydrogenizes the resulting isocitrate to yield α-ketoglutarate (α-KG), which converts to succinyl-CoA by the catalysis of α-ketoglutarate dehydrogenase. The succinyl-CoA undergoes four steps to produce OAA via the formation of succinate, fumarate, and malate catalyzed by succinyl-CoA synthetase, succinate dehydrogenase, fumarase, and malate dehydrogenase, respectively^11^. Notably, the carboxylic acids in the TCA cycle connect a larger metabolic networks^11^. For example, OAA, fumarate, and α-KG are involved in amino acid biosynthesis/degradation, ammonia assimilation and/or purine nucleotide metabolism/biosynthesis. Malate involves in the glyoxylate cycle and the formation of pyruvate. Moreover, the products of degrading citrate relate to the biosynthesis of γ-aminobutyric acid (GABA)^12^, isoprenoids, flavonoids and fatty acid extension^13^,^14^. In addition, OAA, one of the products of degrading citrate by ATP citrate lyase (ACL, EC4.1.3.8), can reenter the TCA cycle or be utilized for mono- or disaccharide synthesis through the gluconeogenesis pathway, which includes three key enzymes: glucose-6-phosphatase, fructose-1,6-bisphosphatase (FBPase, EC 3.1.3.11), and phosphoenolpyruvate carboxykinase (PEPCK, EC 4.1.1.49)^11^,^15^.

Acidity is important for the fruit’s organoleptic quality. In the citrus juice cell, acidity is generally dependent on citrate accumulation in the cell vacuole where the citrate contributes more than 90% of the total organic acids^8^. Citrate accumulation in the vacuole depends on the balance of citrate synthesis, membrane transport and degradation or utilization^12^,^16^. CS activity may not be responsible for the difference of acidity among citrus varieties^17^,^18^. However, a partial block of mitochondrial Aco (myt-Aco) activity (possibly by citramalate) is the prerequisite for citrate transport into the cell cytoplasm^16^,^19^. When citrate transports into the cytoplasm, vacuolar-type proton pumps play an important role in citrate uptake into the vacuole^20^,^21^,^22^. Also, some p-type proton pumps relate to citrate accumulation in the vacuole^23^,^24^. As the fruit ripens, vacuolar citrate fluxes into the cytoplasm possibly through citrate/H^+^ symporters^25^ and is utilized through the Aco-GABA and/or ACL-degradation pathway(s)^10^,^12^,^26^. Transcript analysis confirmed that the H^+^/citrate symporter CsCit1^25^, the cytosolic Aco (cyt-Aco), cyt-IDH or NADP-IDH, glutamate decarboxylase (GAD, EC 4.1.1.15), and ACL participate in citrate catabolism as the fruit ripens^12^,^16^,^26^,^27^,^28^,^29^,^30^,^31^. Moreover, modifying the process of citrate biosynthesis to utilization can result in a metabolic shift towards amino acid or flavonoid biosynthesis^28^,^32^.

Because the reactions involved in carboxylic acid metabolism are the central point of the biosynthesis and/or catabolic networks of other metabolites, we hypothesized that the comprehensive variation in metabolites in ‘HAL’ compared with ‘AL’ should be tightly related to the changes in citrate metabolism. Hence, we compared the profiles of citrate accumulation-related genes and/or enzyme activities, as well as the metabolic fingerprinting between ‘HAL’ and ‘AL’ in the present study to elucidate the possible mechanism underlying the extensive metabolite changes in ‘HAL’. These results provide a scenario for this mutant and for the investigation of the network of metabolites involved in fruit quality.

## Results

### Differential metabolites between ‘AL’ and ‘HAL’

The high reproducibility of the total ion current of all samples indicated that the raw LC-MS data quality was reliable (Fig. S1). Using the optimized LC-MS analysis protocol and subsequent processes (i.e., raw data conversion, peak alignment and normalization, extraction of the peak m/z value, and retention time), we obtained 1645 features (one m/z value refers to one feature) under positive mode and 1388 features under negative mode. The relative standard deviation (RSD) frequency distributions of group ‘AL’ and group ‘HAL’ were primarily in the 0–30% range under either the positive mode or negative mode (Fig. S2), indicating that the sample deviation in each group was small and that the data quality was acceptable. The principal component analysis (PCA) and partial least squares-discriminant analysis (PLS-DA) confirmed that significant difference in metabolites exists between group ‘AL’ and group ‘HAL’ (Fig. S3). This PCA result explained 57.8% of the variation in the metabolic profiling (R^2^X = 0.578) under positive mode (Fig. S3A) and 58.3% of the variation in the metabolic profiling (R^2^X = 0.583) under negative mode (Fig. S3B). The PLS-DA model achieved a distinct separation between the metabolite fingerprinting of the groups ‘AL’ and ‘HAL’ with R^2^X = 0.463, R^2^Y = 0.994, and Q^2^ = 0.997 under positive mode (Fig. S3C) and R^2^X = 0.459, R^2^Y = 0.997, and Q^2^ = 0.983 under negative mode (Fig. S3D).

The volcano plot visually displayed many features that significantly differed between ‘AL’ and ‘HAL’ (Fig. S4). According to the screening criteria [the variable importance in the projection (VIP) >1 and *p*-value < 0.01], we found 718 and 643 features showing significant difference between ‘AL’ and ‘HAL’ (Table S1) under positive and negative modes, respectively. However, we only identified 33 and 39 metabolites under the positive and negative modes, respectively, by searching the METLIN database (https://metlin.scripps.edu/) and the BGI-Tech local KEGG metabolite databases with the m/z values. The total number of identified metabolites was 68 (Table 1). We further classified these metabolites into seven groups: organic acids, sugars, amino acids and derivatives, purine or pyrimidine nucleosides and analogues, plant hormones and analogues, vitamins and derivatives, and anonymous group (Table 1). In the groups of organic acid and sugars, pyruvic acid, citric acid, oxoglutaric acid, isocitric acid, and hexose 1-phosphate had significantly lower levels in ‘HAL’ compared with ‘AL’; their levels in ‘HAL’ were approximately half or less of their levels in ‘AL’. Conversely, the succinic acid, fumaric acid, malic acid, citramalic acid, sucrose, sucrose 6-phosphate, D-glucose 6-phosphate, and 3-O-alpha-L-arabinopyranosyl-L-arabinose contents were significantly higher in ‘HAL’ than ‘AL’. In particular, the succinic acid, sucrose, and 3-O-alpha-L-arabinopyranosyl-L-arabinose contents in ‘HAL’ were 2-fold higher than those in ‘AL’. In the amino acids and derivatives group, the L-lysine and histidinol phosphate levels were significantly lower in ‘HAL’ than in ‘AL’, whereas the histidine, serine, threonine, pyrroline hydroxycarboxylic acid, and phenylpyruvic acid levels were significantly higher in ‘HAL’ than in ‘AL’. Notably, the L-lysine level in ‘HAL’ was less than one-tenth the level in ‘AL’, whereas the histidine level under negative mode and the pyrroline hydroxycarboxylic acid level under positive mode were more than 2- and 4-fold higher in ‘HAL’ than in ‘AL’, respectively. In the purine or pyrimidine nucleosides and analogues groups, all identified metabolites, including guanosine, deoxyinosine, uridine, uracil, and hypoxanthine, had significantly higher levels in ‘HAL’ compared with ‘AL’. Specifically, the levels of uridine and deoxyinosine were more than 6- and 28-fold higher in ‘HAL’ than in ‘AL’, respectively. In the plant hormones and analogues group, abscisic acid (ABA), abscisic alcohol 11-glucoside, and methyl jasmonate (MeJA) showed significantly lower levels in ‘HAL’ compared with ‘AL’, whereas the levels of zeatin analogues and dihomo-jasmonic acid were significantly higher in ‘HAL’ than in ‘AL’. In the vitamins and derivatives group, four types of metabolites exhibited significant differences between the two cultivars. In them, the levels of ascorbic acid, L-ascorbic acid-2-glucoside, and niacin were nearly 2-fold higher in ‘HAL’ than in ‘AL’, whereas the riboflavin content in ‘HAL’ was less than half of the riboflavin content in ‘AL’. In addition, we clustered 32 identified metabolites into the anonymous group due to their complex or unclear functions. In them, 15 metabolites had significantly lower levels and 17 metabolites had significantly higher levels in ‘HAL’ compared with ‘AL’. The isopentenyl adenosine-5′-diphosphate, propyl cinnamate, and limonexic acid contents in ‘HAL’ were one-fourth or less of the contents in ‘AL’, whereas the levels of rhamnocitrin 3-(6′-acetylglucoside), methyl pentenoic acid, and cinnamic acid were increased by 3.5-fold and the octenoic acid level was increased by 20-fold in ‘HAL’ compared with ‘AL’.

### Comparative analysis of citrate biosynthesis-related enzymes

**Figure 1:**
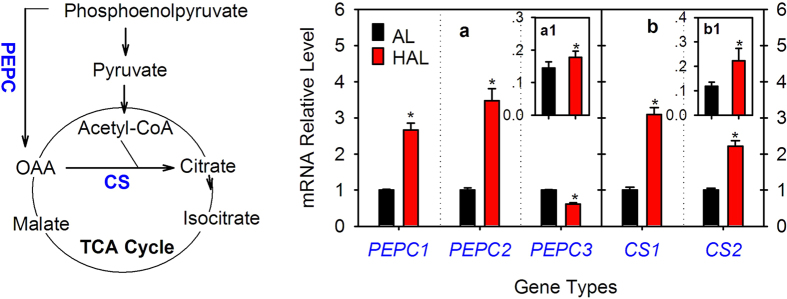
Gene expression and activity analysis of citrate biosynthesis-related enzymes. PEPC, OAA, CS, AL and HAL are the abbreviations of phosphoenolpyruvate carboxylase, oxaloacetate, citrate synthase, ‘Anliu’ orange and ‘Hong Anliu’ orange, respectively. (**a**) and (**b**) refer to the results of the PEPC and CS gene expression analysis, respectively. a1 and b1 refer to the activities (U·min^−1^·g^−1^FW) of the PEPC and CS enzymes, respectively. The asterisks (*) on the bars indicate significant differences (*P* < 0.05) between the AL and HAL fruits in the *t-*tests (LSD).

CS catalyzes the condensation of acetyl-CoA and OAA to form citrate and PEPC catalyzes the β-carboxylation of phosphoenolpyruvate to produce OAA. CS is directly responsible for citrate synthesis, whereas PEPC has been suggested to influence citrate biosynthesis^33^. Although one PEPC gene and one CS gene were previously cloned from citrus^17^,^18^,^33^, we further screened the citrus genome databases and the PCR confirmation indicated that there were at least three PEPC and two CS gene members in the current citrus genome databases (Table S2, Fig. S5A,B). Then, we analyzed their expression levels in both the ‘AL’ and ‘HAL’ fruit juice sacs at 235 days after florescence (DAF) (Fig. 1a,b). The *PEPC*1 and *PEPC*2 transcript levels were significantly higher in ‘HAL’ than ‘AL’, whereas the *PEPC*3 transcript level in ‘HAL’ was markedly lower than that in ‘AL’ (Fig. 1a). In contrast to the three PEPC genes, the transcript levels of the two CS genes were both significantly higher in ‘HAL’ than ‘AL’ (Fig. 1b). We further analyzed their enzyme activities and found that the PEPC (Fig. 1a1) and CS (Fig. 1b1) activities were significantly higher in ‘HAL’ than ‘AL’.

### Comparative analysis of citrate transport-related genes

**Figure 2:**
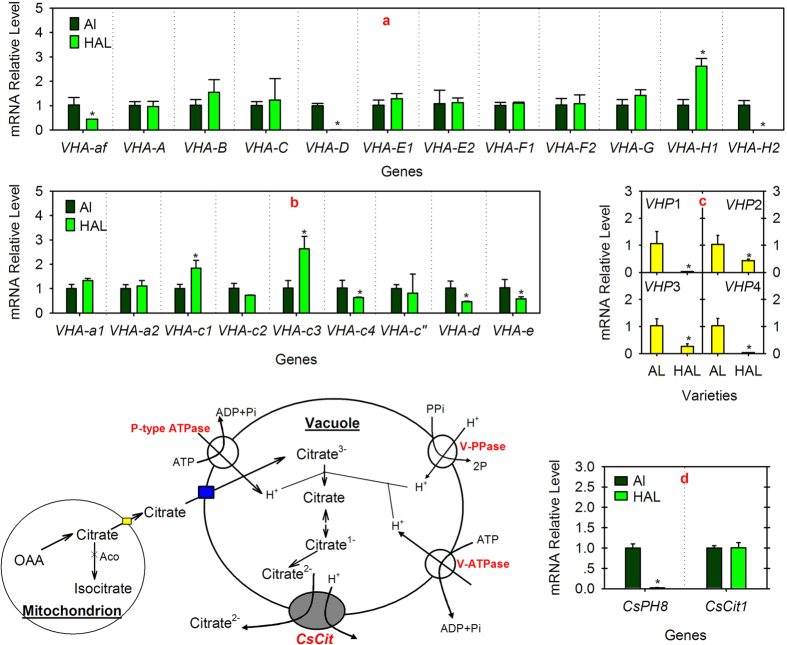
Expression profiles of the genes involved in citrate transport. (**a,b**) refer to the expression profiles of the genes encoding the VHA assembly factor (*af*) or different VHA subunits, respectively (**c**) refers to the expression profiles of the genes encoding VHP (**d**) refers to the expression profiles of *CsPH8* (Shi *et al*.^23^) and *CsCit1* (Shimada *et al*.^25^). The asterisks (*) on the bars indicate significant differences (*P* < 0.05) between the AL and HAL fruits in the *t-*tests (LSD).

The expression profiles of the genes encoding vacuolar H^+^-ATPase (VHA), the vacuolar H^+^-PPase (VHP), *CsPH8* encoding the P-type proton pump^23^ and *CsCit1*^25^ were compared between ‘AL’ and ‘HAL’ (Fig. 2).

VHA consists of a peripheral V1 domain and a membrane-integral V0 domain. VHA contains 13 subunits (VHA-A, VHA-B, VHA-C, VHA-D, VHA-E, VHA-F, VHA-G, and VHA-H for the V1 domain and VHA-a, VHA-c, VHA-c”, VHA-d, and VHA-e for the V0 domain); moreover, there is an assembly factor (af) for VHA assembly^34^. Through screening the citrus genome databases and PCR confirmation, we found at least one gene encoding af, VHA-A, B, C, D, G, c”, d, and e, two genes encoding VHA-E, F, H, and a, and four genes encoding VHA-c (Table S2, Fig. S5F–H). Transcript analysis indicated that the *VHA-af* transcript level of ‘HAL’ was half that of ‘AL’ (Fig. 2a). Moreover, the transcript levels of some genes (i.e., *VHA-D*, *VHA-H2*, *VHA-c4*, *VHA-d*, and *VHA-e*) were extremely or significantly lower, whereas the transcript levels of *VHA-H1*, *VHA-c1*, and *VHA-c3* were significantly higher in ‘HAL’ than ‘AL’. Additionally, the transcript levels of other genes encoding the VHA V1 and V0 domains showed no significant difference between the two cultivars (Fig. 2a,b). VHP is another V-type proton pump^35^. Inquiry of the citrus genome and PCR confirmation indicated the presence of at least four genes (*VHP1-4*) encoding VHP (Table S1 and Fig. S5I). Transcript analysis showed that the transcript levels of *VHP1-4* were significantly lower in ‘HAL’ than ‘AL’ (Fig. 2c). Similar to *VHP1-4*, the *CsPH8* transcript level was also significantly lower in ‘HAL’ than ‘AL’. However, the *CsCit1* transcript level had no significant difference between ‘HAL’ and ‘AL’ (Fig. 2d).

### Comparative analysis of citrate degradation- or utilization-related genes and/or enzyme activities

Citrate participates in the TCA cycle primarily for energy metabolism in the mitochondria^11^. In the cell cytoplasm, citrate can use for amino acid or GABA biosynthesis through the production of glutamate^12^,^28^, for the biosynthesis of many secondary metabolites and for gluconeogenesis through the ACL-degradation pathway^10^,^13^,^26^,^36^.

**Figure 3:**
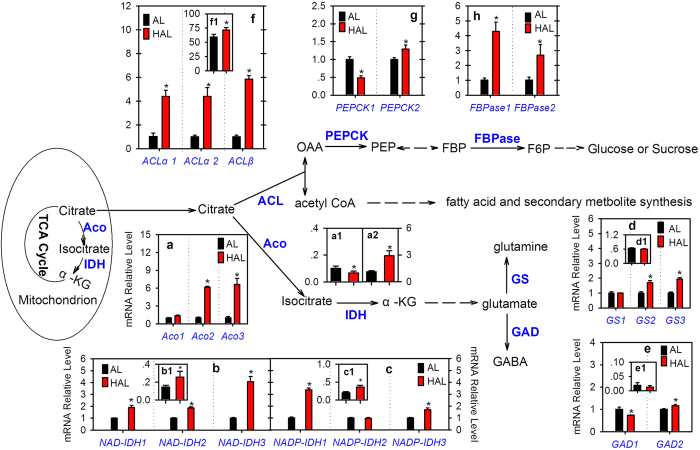
Comparison of citrate degradation- and utilization-related genes expression levels and/or their respective enzyme activities. Aco, IDH, α-KG, GS, GAD, ACL, PEPCK, FBPase, AL and HAL are the abbreviations of aconitase, isocitrate dehydrogenase, α-ketoglutarate, glutamine synthesis, glutamate decarboxylase, ATP-citrate lyase, phosphoenolpyruvate carboxykinase, fructose-1,6-bisphosphatase, ‘Anliu’ orange and ‘Hong Anliu’ orange, respectively. a-f refer to the results of the gene expression analysis, respectively. a1 and a2 refer to the activities (U·min^−1^·g^−1^FW) of mitochondrial Aco and cytoplasmic Aco, respectively. b1 and c1 refer to the activities (U·min·g^−1^FW) of NAD-IDH and NADP-IDH, respectively. d1, e1, and f1 refer to the activities of GS (△OD·h^−1^·g^−1^FW), GAD (GABA mg·h^−1^·g^−1^FW), and ACL (μmol·min^−1^·g^−1^FW), respectively. The asterisks (*) on the bars indicate significant differences (*P* < 0.05) between the AL and HAL fruits in the *t-*tests (LSD).

Aco, IDH, GS and GAD are the key enzymes involved in citrate catabolism through the Aco-GABA pathway (Fig. 3). A previous study indicated that three genes (*Aco1*, *Aco2* and *Aco3*) encode Aco in the citrus genome^29^. Here, transcript analysis showed that the *Aco1* transcript level in ‘HAL’ was slightly higher than that of ‘AL’ and the *Aco2* and *Aco3* transcript levels were more than six-fold higher in ‘HAL’ than in ‘AL’ (Fig. 3a). Enzyme activity analysis showed that the mit-Aco activity was significantly lower (Fig. 3a1) and the cyt-Aco activity was significantly higher (Fig. 3a2) in ‘HAL’ than ‘AL’. IDH is the second key enzyme involved in citrate catabolism. One gene encoding cyt-IDH or NADP-IDH was cloned from citrus by Sadka *et al*.^30^. In this study, inquiry of the citrus genome databases and PCR confirmation showed that at least three NAD-IDH (mitochondrial type) and NADP-IDH (cytoplasmic type) genes exist in the citrus genome (Table S1 and Fig. S5C,D). Transcript analysis indicated that the transcript levels of the three NAD-IDH genes (Fig. 3b) and two NADP-IDH genes (*NADP-IDH1* and *NADP-IDH3*, Fig. 3c) were significantly higher in ‘HAL’ than ‘AL’. Moreover, the enzyme activity analysis showed that the activities of myt-IDH (Fig. 3b1) and cyt-IDH (Fig. 3c1) were both significantly higher in ‘HAL’ than ‘AL’. GS and GAD are two enzymes that catalyze glutamate in the cytoplasm (Fig. 3). Three genes (*GS1*-*3*) encoding GS were confirmed in the citrus genome (Table S1 and Fig. S5B) and two genes (*GAD1*-*2*) were previously confirmed by Liu *et al*.^31^. Transcript analysis indicated that the transcript levels of *GS2* (Fig. 3d), *GS3* (Fig. 3d) and *GAD2* (Fig. 3e) were significantly higher in ‘HAL’ than ‘AL’. Differently, the *GS1* transcript level was similar between ‘HAL’ and ‘AL’ (Fig. 3d) and the *GAD1* transcript level was significantly lower in ‘HAL’ than ‘AL’ (Fig. 3e). Furthermore, the enzyme activities of GS and GAD showed similar levels between ‘HAL’ and ‘AL’ (Fig. 3d,e).

ACL catalyzes the cleavage of citrate to yield acetyl-CoA and OAA, which involve in the biosynthesis of many secondary metabolites or gluconeogenesis (Fig. 3). Three ACL genes have been confirmed in the citrus genome^26^. Here, transcript analysis showed that the transcript levels *of ACLα*1, *ACLα*2 and *ACLβ* were more four-fold higher in ‘HAL’ than ‘AL’ (Fig. 3f). Moreover, the ACL enzyme activity in ‘HAL’ was significantly higher than that in ‘AL’ (Fig. 3f1).

The PEPCK and FBPase are the key enzymes involving in gluconeogenesis^11^,^15^. The sequence inquiry and PCR confirmation indicated that at least two genes encoding PEPCK and FBPase exist in the citrus genome (Table S1, Fig. S5A,E). The *PEPCK1* transcript level was significantly lower and the *PEPCK2* transcript level was significantly higher in ‘HAL’ than ‘AL’ (Fig. 3g). Differently from the PEPCK genes, the transcript levels of the *FBPase1* and *FBPase2* genes were both significantly higher in ‘HAL’ than ‘AL’ (Fig. 3h).

## Discussion

‘HAL’ is a bud mutant of the ‘AL’ sweet orange^1^. It is characterized by a comprehensive alteration in metabolites, such as the lycopene, the soluble sugars (sucrose, fructose and glucose), the organic acids and other secondary metabolites^1^,^2^,^4^. To help investigating the possible reason for the comprehensive change, we analyzed the metabolites in the fruits of the two cultivars at 242 DAF by using LC-Q/TOF-MS technique in the present study. Although we found more than 600 features showing significant difference between the two cultivars under the current analysis conditions (Table S1), only approximately one-tenth of the different features were identified (Table 1). Nevertheless, the profiles of differently identified metabolites between the two cultivars were almost consistent with the report of Pan *et al*.^2^, implying that the data is believable.

As the carboxylic acid metabolism is the central point of the biosynthesis and/or catabolic networks of other metabolites, we further assessed the significance of citrate metabolism in the comprehensive metabolite alterations in ‘HAL’. Clearly, citrate is significantly lower in the juice sacs of ‘HAL’ than ‘AL’ during fruit development and ripening^1^,^4^, which was further confirmed in the present study (Table 1). However, we found that the transcript levels of citrate biosynthesis-related genes (*CS*1, *CS*2, *PEPC*1, *PEPC*2) and the enzyme activities of CS and PEPC were significantly higher in ‘HAL’ than ‘AL’ (Fig. 1), indicating that ‘HAL’ has an increased citrate biosynthesis ability compared with ‘AL’. Thus, we can deny the hypothesis that the low accumulation of citrate in ‘HAL’ is possibly due to the reduction of citrate biosynthesis.

It is well known that a partial block of myt-Aco activity is a prerequisite for citrate transport into the cell cytoplasm^16^,^19^. Bogin and Wallace^19^ first suggested that the citramalate was possibly the inhibitor of myt-Aco. Degu *et al*.^28^ found that spraying citramalate did inhibit Aco activity and increased citrate accumulation in citrus fruit juice sacs. Consistent with the study of Pan *et al*.^2^, we found that the citramalate or citramalic acid content was significantly higher (1.24-fold) in ‘HAL’ than ‘AL’ (Table 1), moreover, the myt-Aco activity was significantly lower in ‘HAL’ than ‘AL’ (Fig. 3a1). These results implied that more citrate in the mitochondrion transports into the cytoplasm in ‘HAL’ than ‘AL’.

When citrate transports into the cytoplasm, it should utilize immediately or store quickly in the vacuole to keep the cytoplasm neutral for cell activity^15^,^37^. The uptake of citrate into the vacuole depends on the activity of the proton pumps^21^,^22^ and the utilization of citrate depends on the activities of enzymes involved in Aco-GABA pathway^12^ and/or ACL-pathway^26^. Here, we found that some key VHA genes (Fig. 2a,b) [e.g., the VHA assembly factor gene (*VHA-af*), all the VHP genes (Fig. 2c), and the juice sac-predominant p-type proton pump gene (*CsPH*8)^23^] showed significantly lower expression levels in ‘HAL’ than ‘AL’. On the other hand, the activities of cyt-Aco and NADP-IDH in the Aco-GABA pathway and ACL in the ACL-degradation pathway were significantly higher in ‘HAL’ than ‘AL’ (Fig. 3). These results indicated that in the ‘HAL’, more citrate does not accumulate in the vacuole but utilizes in the cytoplasm because the lower expression levels of proton pump genes reduce the ability of the proton pump to uptake citrate into the vacuole. Moreover, the higher enzyme activities of cyt-Aco, NADP-IDH and ACL increase the ability to utilize citrate.

The TCA cycle is very important for organisms by generating energy and providing various precursors for other biochemical reactions^11^. The present study showed that the citramalate content increased significantly (Table 1) and the myt-Aco activity reduced clearly (Fig. 3a1), suggesting that the TCA cycle should be seriously blocked in ‘HAL’ compared with ‘AL’. However, we also found that the cyt-Aco (Fig. 3a2) and NADP-IDH (Fig. 3c1) activities increased significantly in ‘HAL’ compared with ‘AL’ and the activities of GS (Fig. 3d1) and GAD (Fig. 3e1) of which both enzymes catalyze glutamate showed similar levels between ‘HAL’ and ‘AL’. These results suggested that the direct products (isocitrate and α-KG) of cyt-Aco and NADP-IDH should be significantly higher in ‘HAL’ than ‘AL’. Nevertheless, they decreased significantly in ‘HAL’ compared with ‘AL’ (Table 1). Therefore, we inferred that more cytoplastic isocitrate and α-KG in ‘HAL’ import into the mitochondrion and participate in the TCA cycle again. The compensation of isocitrate and α-KG from the catalysis of cytoplastic citrate by cyt-Aco and NADP-IDH contributes to the stability of the TCA cycle in ‘HAL’.

**Figure 4:**
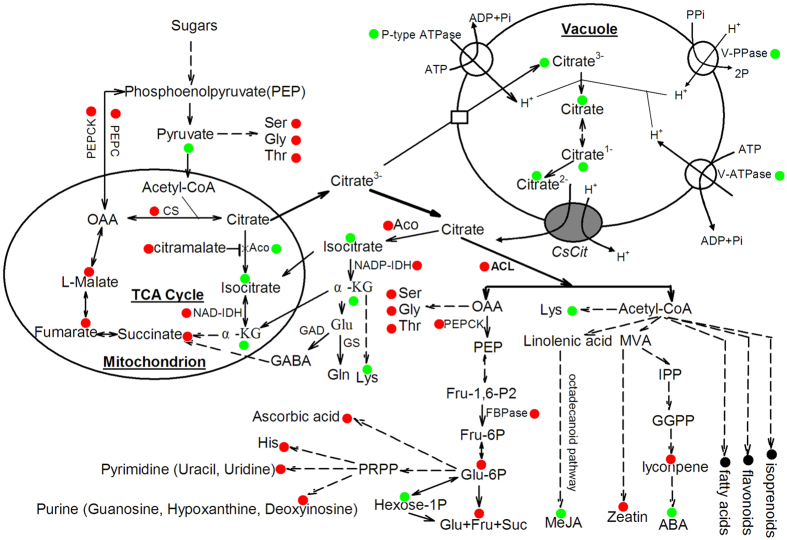
Overview of the major metabolic pathways involved in mutant trait formation. The red circle indicates that the metabolite content, enzyme activity or gene expression level is significantly higher in the mutant cultivar (‘HAL’) than the wild type cultivar (‘AL’). The green circle indicates that the metabolite content, enzyme activity or gene expression level is significantly lower in ‘HAL’ than ‘AL’. The black circle indicates that the difference of other metabolites between ‘HAL’ and ‘AL’ is not clear.

Another cytoplastic citrate-degrading pathway is through ACL catalysis, which links the production of many secondary metabolites^13^,^36^. For example, Crifò *et al*.^32^ suggested that citrate could utilize for flavonoid biosynthesis through ACL catalysis in blood oranges under cold storage. In the present study, the ACL gene transcript levels (Fig. 3f) and enzyme activity (Fig. 3f1) were significantly higher in ‘HAL’ than ‘AL’. These findings indicated that ACL is more active in the low-acid orange ‘HAL’ than in the normal-acid orange ‘AL’, implying that more citrate is cleaved to produce more OAA and acetyl-CoA. OAA can reenter into the mitochondrion for citrate biosynthesis or use for gluconeogenesis or amino acid biosynthesis^11^,^15^. Here, we found that the transcript levels of three key genes [*PEPCK2* (Fig. 3b), *FBPase1* and *FBPase2* (Fig. 3h)] involving in gluconeogenesis, the intermediate sugar (Glu-6P) and the soluble sugar (Suc, Glu, and Fru) contents were significantly higher in ‘HAL’ than in ‘AL’ (Table 1). These results indicated the OAA from the catalysis of cytoplastic citrate by ACL participates in the gluconeogenesis, which at least accounts partially for the significant increase of the soluble sugars in ‘HAL’. Judging from the Fig. 4, the significant increase of Glu-6P at least contributes partially to the significantly increase of ascorbic acid, histidine (His), pyrimidine or purine in ‘HAL’ compared with ‘AL’ (Table 1). In addition, the contents of serine, glycine and tryptophan were significantly higher in ‘HAL’ than ‘AL’ (Table 1), which is possibly related to the participation of OAA or pyruvate (Fig. 4). On the other hand, another product of acetyl-CoA via ACL catalysis usually utilizes for fatty acid extension or the biosynthesis of isoprenoids and other secondary metabolites^13^,^32^,^36^, including the biosynthesis of lysine (Lys), methyl jasmonate (MeJA), zeatin, lycopene or ABA (Fig. 4). The present study showed that the content of zeatin was significantly higher but the contents of Lys, MeJA and ABA were significantly lower in ‘HAL’ than ‘AL’ (Table 1), consistent with the previous study of Pan *et al*.^2^. In addition, we are also sure that lycopene is significantly higher in ‘HAL’ than ‘AL’ because lycopene can be visibly found in ‘HAL’ (Fig. S6) and has been detected by HPLC at a significant level in ‘HAL’^1^,^4^. In this study, consistent with a previous metabolomics analysis 2, we failed to identify lycopene in ‘HAL’ possibly due to the lack of a standard reference database for citrus fruits or just using a colomn C18 not a C30 in the LC-MS analysis. Furthermore, we believe that there should have more significantly altered secondary metabolites in ‘HAL’ because 90% of the differential features failed identification in this study (Table S1 and Table 1). This failure is largely due to our basic lack of knowledge of many metabolic pathways in plants and our current inability to identify the majority of metabolites synthesized in the citrus plant.

Taken together, this study confirmed that the orange mutant ‘HAL’ has a comprehensive alteration in metabolites except for the increase in lycopene and soluble sugars and the decrease in citrate. Combining the present and previous results^1^,^2^,^3^,^4^,^6^, we established a model to explain the comprehensive alteration in metabolites in the orange mutant ‘HAL’ (Fig. 4). In detail, although the citrate content is very low in ‘HAL’ juice sacs, ‘HAL’ still has higher ability to biosynthesize the citrate comprared with ‘AL’. Moreover, the citramalate content (the inhibitor of mit-Aco) increases significantly and the mit-Aco activity decreases significantly in ‘HAL’, resulting in more synthesized citrate exports into the cytoplasm. Because the transcript levels of some key genes of proton pump decreases significantly in ‘HAL’ compared with ‘AL’, which reduces the ability for the uptake of citrate into the vacuole, more citrate cannot store in the vacuole. The abundant citrate utilize immediately because the activities of cys-Aco, NADP-IDH and ACL increase significantly in the ‘HAL’ compared with the ‘AL’, which keeps the cytoplasmic pH constant. Some citrate-splitting products possibly reenter the mitochondrion to maintain the stability of the TCA cycle rather than participate in the biosynthesis of GABA or glutamine. The others utilize for gluconeogenesis and/or secondary metabolite metabolism, which results in a significant increase in the soluble sugars, ascorbic acid, histidine, pyrimidine, purine, zeatin, and lycopene or a significant decrease in methyl jasmonate and ABA (Fig. 4).

Frankly, the model gives a rough but reasonable explanation for the comprehensive change in metabolites of ‘HAL’ compared with its wild type, ‘AL’. In the model, the decrease of proton pump genes’ expression, namely, the reduction of proton pump ability to promote the citrate storing in the vacuole plays a pivotal role for the comprehensive alteration in metabolites of ‘HAL’. Although the lower ABA content in the ‘HAL’ compared with ‘AL’ is possibly due to the higher accumulation of lycopene in ‘HAL’, which always reduces ABA biosynthesis^1^,^38^, there is a long way to verify this model in the future because of the complexity in the secondary metabolism and the difficulty for gene function identification of perennial plants.

## Methods

### Plant materials and sample preparation

The ‘Anliu’ sweet orange (AL, *Citrus sinensis* cv. Anliu) and its mutant ‘Hong Anliu’ (HAL, *C. sinensis* cv. Hong Anliu) grafted onto the same rootstock (trifoliate orange as base rootstock and Satsuma mandarin as middle rootstock) at the citrus germplasm orchard in Huazhong Agricultural University (Hubei province, China) were used in the present study (Fig. S6). We collected ten nearly uniform fruits of each cultivar randomly at 235 DAF for the gene expression and enzyme activity analyses. Because the metabolite response occurs later than the gene reaction, we collected another ten fruits collected at 242 DAF (seven-day interval) for the metabolite analysis. The fruit segments were separated immediately, frozen in liquid nitrogen and stored at −80 °C prior to use.

### LC-MS analysis

Ten fruits (n = 10) of ‘AL’ and ‘HAL’, respectively at 242 DAF were sent to BGI TechSolutions Co., Ltd. (BGI-Tech, Shenzhen, China) for metabolite analysis. One segment was randomly selected from each fruit and ground into powder in liquid nitrogen. The powder was ultrasonically oscillated with 800 μL of solvent (acetonitrile:water = 7:3) for 20 minutes. A total of 300 μL of homogenate was transferred into a clean Eppendorf tube and centrifuged for 10 min at 12,000 × g and 4 °C. A total of 100 μL of the supernatant was used for the sample injection.

The detection instrument was the LC-Q/TOF-MS (Agilent, 1290 Infinity LC, 6530 UHD and Accurate-Mass Q-TOF/MS) with a C_18_ chromatographic column (Agilent, 100 mm × 2.1 mm, 1.8 μm). The separation conditions were as follows: column temperature, 40 °C; flow rate, 0.35 mL/min; pressure limit, 800 bar; mobile phase A, 0.1% formic acid in water (v/v); mobile phase B, 0.1% formic acid in acetonitrile (v/v); and injection volume and temperature, 4 μL and 4 °C. The elution gradient was 5% mobile phase B at 0 and 1 min, 20% at 6 min, 50% at 9 min, and 95% at 13 and 15 min.

Sample analysis performed under the positive and negative ion modes and their mass parameters showed in Table S3. Nitrogen was as the cone or desolvation gas. The ion scan time was 0.03 s with a scan interval of 0.02 s. The mass scanning range was 50–1000 m/z. The data were processed with the standard procedure^39^,^40^.

### Real-time PCR expression analysis

Total RNAs of the fruit segment samples at 235 DAF were isolated from with the RNAprep Pure Plan Kit (TIANGEN BIOTECH CO., LTD., Beijing, China) according to the manual protocol. One μg of high-quality total RNA was used for first-strand cDNA synthesis using the PrimeScript RT Reagent kit with gDNA Eraser (TaKaRa, DALIAN, China). The citrate-accumulated genes used here included three *PEPC*s, two *CS*s, one *CsCit1*^25^, three *Aco*s^29^, three *NAD-IDH*s, three *NADP-IDH*s, three glutamine synthetases (EC 6.3.1.2) (*GS*s), two GADs^31^, three ACLs^26^, one p-type proton pump gene (*CsPH*8)^23^, twenty-four V-type proton pumps, and an assembly factor (af) for vacuolar H^+^-ATPase (VHA) assembly (*VHA-af*). Moreover, two *PEPCK*s and *FBPase*s involved in gluconeogenesis were included. Their sequences were identified from the citrus genome databases (citrus.hzau.edu.cn/orange/and phytozome.jgi.doe.gov/pz/portal.html) followed by PCR confirmation or referenced from other studies (Table 2, Table S2 and Fig. S5). The authenticity of the sequences from the citrus genome databases confirmed by PCR amplification using ‘AL’ fruit cDNA as the template prior to primer design for the quantitative real-time PCR (qRT-PCR). Primers specific for the targeted genes and the actin gene were designed with Primer 3.0^41^ and listed in Table 2 or Table S2. The qRT-PCR performed in a 10-μL reaction volume using SYBR *Premix Ex Taq* (TaKaRa, DALIAN, China) on a LightCycler 480 Real-Time System according to the manufacturer’s protocol. The qRT-PCR carried out for three biological replicates. The reactions started with an initial incubation at 50 °C for 2 min, followed by 95 °C for 10 min and 40 cycles of 95 °C for 15 s and 60 °C for 60 s. The Livak method^42^ was employed to calculate the relative gene expression level.

### Enzyme activity determination

The determination of each enzyme activity performed in triplicate. The activities of CS, cyt- and myt-ACO, and cyt- and myt-IDH were assayed with the methods reported by Luo *et al*.^43^ and Hirai and Ueno^44^. The ACL activity was analyzed with the method described by Hu *et al*.^26^, and the GAD activity was assayed with the method described by Liu *et al*.^31^.

Glutamine synthetase (GS, EC 6.3.1.2) activity was assayed with the method described by Kaiser and Lewis^45^ with modifications. Two grams of sample were ground into powder with liquid nitrogen. Then, the powder was homogenized with 4 ml of cold extraction buffer (0.1 mM phosphate buffer containing 1 mM EDTA, 2 mM dithiothreitol and 8% insoluble polyvinylpyrrolidone), incubated on ice for 10 min, and centrifuged at 12,000 × g for 15 min at 4 °C. The supernatant stored at 0–4 °C and used as the crude enzyme solution. The enzyme assay was the same as that described by Kaiser and Lewis^45^, and the absorbance was measured at 540 nm.

### Data processing and statistics

Similar with other reports^2^,^46^, the LC-MS raw data were initially converted into the netCDF format and then processed by the XCMS toolbox (http://metlin.scripps.edu/xcms/)^47^. After m/z data normalization, the data quality was evaluated by calculating the relative standard deviation (RSD) and drawing the RSD histogram. The data was pre-processed by both mean-centering and variance-scaling prior to multivariate analysis. Then, the resulting scaled datasets were imported into simca-p software (Version 12.0, Umetrics, Umea, Sweden) and the unsupervised PCA and supervised PLS-DA were carried out to test the difference of metabolomic data between groups of ‘AL’ and ‘HAL’. The significantly different features were screened out with the combination of VIP value (>1) from PLS-DA and the p value (<0.01) from a two-tailed Student’s t-test. The differential m/z candidates were putatively identified through searching the online Metlin database (metlin.scripps.edu/)^48^ and the local KEGG databases (version 59, BGI TechSolutions Co., Ltd., Shenzhen, China).

On the other hand, the significant difference of gene expression or enzyme activity between ‘AL’ and ‘HAL’ at 235 DAF was evaluated with Student’s t test in the ANOVA program of SAS (SAS Institute, Cary, NC, USA). Difference was considered significant at P < 0.05.

## Additional Information

**How to cite this article**: Guo, L.-X. *et al*. Citrate Accumulation-Related Gene Expression and/or Enzyme Activity Analysis Combined With Metabolomics Provide a Novel Insight for an Orange Mutant. *Sci. Rep.* **6**, 29343; doi: 10.1038/srep29343 (2016).

## Supplementary Material

Supplementary Information

## Figures and Tables

**Table 1:** Classification of the putatively identified metabolites that were significantly different between ‘HAL’ (M) and ‘AL’ (W).

Classification	Putative Name	m/z	Retension time (min)	Fold change (M/W)	Detection mode
Organic acids	Pyruvic acid	111.00535	1.028	0.24	POSITIVE
	Citric acid	193.03266	1.032	0.21	POSITIVE
	Citric acid	191.02135	1.029	0.49	NEGATIVE
	Oxoglutaric acid	147.02684	1.025	0.26	POSITIVE
	Isocitric acid	191.02135	1.029	0.49	NEGATIVE
	Succinic acid	117.02051	1.404	2.21	NEGATIVE
	Fumaric acid	115.00441	0.923	1.34	NEGATIVE
	Malic acid	133.01496	0.925	1.32	NEGATIVE
	Citramalic acid	147.03118	1.410	1.24	NEGATIVE
Sugars	Hexose 1-phosphate	241.0117	0.726	0.43	POSITIVE
	D-Glucose 6-phosphate	259.0243	0.730	1.43	NEGATIVE
	Sucrose 6-phosphate	423.08989	5.560	1.67	NEGATIVE
	3-O-alpha-L-Arabinopyranosyl-L-arabinose	845.27875	0.839	2.31	NEGATIVE
	Sucrose	342.10781	2.830	2.59	NEGATIVE
Amino acids and derivatives	L-Lysine	147.11607	8.394	0.07	POSITIVE
	Histidinol phosphate	222.06526	6.738	0.56	POSITIVE
	Threonine	118.05209	0.786	1.45	NEGATIVE
	Serine	104.03656	0.774	1.52	NEGATIVE
	Pyrroline hydroxycarboxylic acid	130.0496	1.207	1.66	NEGATIVE
	Phenylpyruvic acid	165.05436	5.136	1.79	POSITIVE
	Histidine	154.06336	0.752	2.07	NEGATIVE
	Pyrroline hydroxycarboxylic acid	128.03637	1.220	4.51	POSITIVE
Purine or pyrimidine nucleosides and analogues	Uracil	113.0344	1.207	1.41	POSITIVE
	Uridine	245.07651	1.210	1.44	POSITIVE
	Guanosine	284.09683	1.048	1.56	POSITIVE
	Hypoxanthine	135.03158	0.810	1.75	NEGATIVE
	Uridine	243.06432	1.222	6.08	NEGATIVE
	Deoxyinosine	251.07958	0.995	28.27	NEGATIVE
Plant hormones and analogues	Abscisic Acid	265.1434	7.746	0.46	POSITIVE
	Abscisic Acid	263.13068	7.746	0.48	NEGATIVE
	Abscisic alcohol 11-glucoside	411.20525	8.60	0.51	NEGATIVE
	Methyl jasmonate	223.13491	7.394	0.67	NEGATIVE
	trans-Zeatin/Pantothenic Acid	220.11792	2.998	1.32	POSITIVE
	trans-Zeatin/Pantothenic Acid	218.10499	2.994	1.45	NEGATIVE
	Cis-zeatin-O-glucoside	380.15868	3.28	1.68	NEGATIVE
	dihomo-jasmonic acid	237.15145	8.615	1.83	NEGATIVE
Vitamins and derivatives	Ascorbic acid	175.02559	0.933	1.90	NEGATIVE
	L-Ascorbic acid-2-glucoside	337.08065	0.908	1.99	NEGATIVE
	Niacin	124.03601	1.041	1.76	POSITIVE
	Riboflavin	375.13283	5.17	0.48	NEGATIVE
Anonymous group	Isopentenyl adenosine-5′-diphosphate	494.09674	7.45	0.24	NEGATIVE
	Propyl cinnamate	191.10609	3.185	0.25	POSITIVE
	Limonexic acid	503.19124	7.487	0.25	POSITIVE
	alpha-Methylstyrene	119.0854	7.390	0.42	POSITIVE
	Inositol cyclic phosphate	241.0117	0.726	0.43	NEGATIVE
	Ambolic acid	471.38581	0.674	0.45	POSITIVE
	Ethylphenol/Dimethylphenol	123.08023	7.392	0.45	POSITIVE
	Nicotinate D-ribonucleoside	256.07836	7.393	0.46	POSITIVE
	Pyridoxamine-5′-Phosphate	249.0607	7.390	0.49	POSITIVE
	L-Citronellol glucoside	317.19884	7.044	0.50	NEGATIVE
	Furoic acid	111.00918	1.029	0.52	NEGATIVE
	Ubiquinone-1	251.12784	7.057	0.54	POSITIVE
	Propylphenol	137.09579	6.738	0.58	POSITIVE
	Viburtinal	161.05945	4.972	0.63	POSITIVE
	S-Adenosyl-4-methylthio-2-oxobutanoate	398.1069	8.901	0.65	POSITIVE
	p-Nitroglutethimide	261.08979	5.377	1.34	NEGATIVE
	N-Methylethanolamine phosphate	156.03971	0.700	1.44	POSITIVE
	Citraconic acid	129.02042	1.045	1.48	NEGATIVE
	Ascorbalamic acid	262.0594	0.826	1.48	NEGATIVE
	p-Coumaroyl quinic acid	339.10751	7.303	1.56	POSITIVE
	Caffeic acid 3-O-glucuronide	355.06987	3.773	1.61	NEGATIVE
	Ethyl (S)-3-hydroxybutyrate glucoside	293.1259	5.013	1.62	NEGATIVE
	1-Linoleoylglycerophosphocholine	520.33997	11.871	1.77	POSITIVE
	Glucocaffeic acid	341.09185	4.189	1.88	NEGATIVE
	Phenprocoumon	279.11064	3.206	1.98	NEGATIVE
	DHAP(8:0)	295.09632	1.470	2.53	NEGATIVE
	Biocytin	104.03656	0.774	2.68	NEGATIVE
	Limocitrin 3-rutinoside	655.19317	8.047	2.69	POSITIVE
	Rhamnocitrin 3-(6′-acetylglucoside)	505.1321	7.805	3.53	POSITIVE
	Methyl pentenoic acid	115.07536	4.405	3.88	POSITIVE
	Cinnamic acid	149.05952	4.721	5.72	POSITIVE
	Octenoic acid	143.10642	8.313	20.31	POSITIVE

**Table 2:** The primers used for qRT-PCR.

Category	Gene Name	Description	Sequence (5′-3′)		Sequence ID* or reference
			Forward primer	Reverse primer	
Citrate synthesis-related	*PEPC1*	phosphoenolpyruvate carboxylase	GTGCGATCCCGTCTATCTGT	AAGGCTCAAGGCCACTTTTT	orange1.1g002089m
	*PEPC2*		GGCATGCAAAACACTGGTTA	CATGTTCATTACGGCTTGGA	orange1.1g002112m
	*PEPC3*		GAACAATGACGGACACAACG	TGGACTCGCTTCCAACTTCT	orange1.1g001537m
	*CS1*	citrate synthase	GGTGCCCCCAATATTAACAA	AGAGCTCGGTCCCATATCAA	orange1.1g012107m
	*CS2*		ACTGGTGTATGGATGCGACA	TCTTCGTCTTGTGGCATTTG	orange1.1g010304m
Citrate degradation or utilization-related	*Aco1*	aconitase	GGCAAGTCATTCACATGCGTT	TGAAGAAGTAGACCCCGGTTGA	Terol *et al*.^29^
	*Aco2*		GGCAATGATGAAGTGATGGCT	GTTGGAACATGGACCGTCTTT	
	*Aco3*		TGCAGCAATGAGGTACAAGGC	TCACACCCAGAAGCATTGGAC	
	*ACLα1*	ATP-citrate α subunit	GATACTGTTGGAGACTTGGG	GCTCTCTTACGACCATCAGG	Hu *et al*.^26^
	*ACLα2*		TACAGTGGAGCACCCAACGA	CCTTCAGGGCTTGGATTATG	
	*ACLβ*	ATP-citrate β subunit	GAGGAGATAACAGAGACAAA	AACAAAGAGCCCATTCAGAT	
	*NAD-IDH1*	NAD-isocitrate dehydrogenase	TATTGCTGGAGGCACTGGTG	ACTTCCCCTCTGCAATTGTG	orange1.1g018224
	*NAD-IDH2*		CAGCACCTGATATTGCTGGA	CTCTGCAATTGTGCTCAGGA	Ciclev10025889m
	*NAD-IDH3*		AGCAGGAAACGTGGGTAATG	GGCAGCAATAACAGCATCAA	orange1.1g017413
	*NADP-IDH1*	NADP-isocitrate dehydrogenase	GAAAATTGGGGATTGGGATT	CAACAGAGGTGCAGCTCAAA	orange1.1g015012
	*NADP-IDH2*		CAGCGGACATGTGAACAATC	CCGTCCATTTCAACGATAGG	Ciclev10005058
	*NADP-IDH3*		TACCGGGTTCATCAGAAAGG	AGGCTGCTTCCAGTTTCTCA	orange1.1g009041
	*GS1*	glutamine synthetase	CATCAATGCTATCGCGTGTT	TCTGCATTCTTGGCAGGTTA	orange1.1g013478m
	*GS2*		TTTGGGATGCTCAGTTGTGA	CTGAATGGCTCCCAAAAATG	orange1.1g018391m
	*GS3*		TCAGGATTCACGAGTTCACG	AGCAAAGAACCCACTGTTGC	orange1.1g018434m
	*GAD1*	glutamate decarboxylase	CACCAAAAAGAATGAGGAGACC	CCGTACTTGTGACCACTGACAT	Liu *et al*.^31^
	*GAD2*		ACCGCAATGTGATGGAGAA	GAATTCATCGTGGCGTTTG	
	*PEPCK1*	phosphoenolpyruvate carboxykinase	GGCTACCGAGAATCCAAACA	GTGCTGGGTGTCGATCTCTT	orange1.1g005865m
	*PEPCK2*		GGCTAGCGAAGATTCAAACG	GTCCCTTTAATGGGGGTGTT	orange1.1g006486m
	*FBPase1*	fructose-1,6-bisphosphatase	TGGAAAGCTGAGGCTCTTGT	ACTTCCTCCTGGCTTCCAAT	orange1.1g015111m
	*FBPase2*		TCCCATTTCTGATCAACTTTCC	GCTGAGGAGCAGGCTTTTTA	orange1.1g019437m
Citrate transported-related	*CsCit1*	H^+^/citrate symporter	GTCTCCGTAACAGGCATTGG	ACCACTAAGGGAAGCGTTCA	Shimada *et al*.^25^
	*CsPH8*	p-type proton pump	CCGTGAAGGAATTGATTTGG	CCATGACAATGGATTCCACA	Shi *et al*.^23^
	*VHA-af*	VHA assembly factor	CAGTGCTACTGAACCCTTCTCCTC	ATGCTCTGAATGCTAAATACCCAA	orange1.1g028366m
	*VHA-A*	V-type ATPase A subunit	GATGCCCTTTTCCCTTCAGT	TTTCATTTCCTCGCTCCCCA	Ciclev10030969m
	*VHA-B*	V-type ATPase B subunit	TCAATGTCCTTCCGTCCCTA	TTCTTCTCCGACCACAGCCT	orange1.1g011329m
	*VHA-C*	V-type ATPase C subunit	AAACATTCATTTGACACTCCTCTT	AACTACTCTCTATGCCTGATACCC	Ciclev10015638m
	*VHA-D*	V-type ATPase D subunit	ATTCTTCCTTTGCCCTGATTG	TTTCACATAAGCAGCACGACA	Ciclev10009240m
	*VHA-E1*	V-type ATPase E subunit	CCGTACCGTCTGTCTTTCCTT	ACATCAGCGTCGTTCATTTTC	orange1.1g027450m
	*VHA-E2*		AAGAGTGCTGATTCTCACGAACC	CGAGCAAGAAGGTTCGTGAG	Cs6g10330.1
	*VHA-F1*	V-type ATPase F subunit	ATGGCTGGCAGAGCTCAAAT	CATCTTCAATTGCTTTCACCGTAG	Ciclev10002833m
	*VHA-F2*		GCTTGCTGGAGTTGGGAATG	GCAGGGATCGGCTTGTTATG	Ciclev10022651m
	*VHA-G*	V-type ATPase G subunit	GACTGAGGCAAGCCAAAGAAGA	AGCCCCAGCATTAAGATGATGA	Cs6g11650.2
	*VHA-H1*	V-type ATPase H subunit	CTGTCGCTTGCTTTGATTTGTC	AACCTCGGTATTCTCATGGTTC	orange1.1g034108m
	*VHA-H2*		CAGTGGAGTACTTGGCAACTA	TCCTTCAAACCTTCTTCCAGTTG	Cs7g14520.1
	*VHA-a1*	V-type ATPase a subunit	TGGTAAGAAGAGAGAAGGCTGTAT	CTTGCGAGTTGCTATCAAATGT	orange1.1g003454m
	*VHA-a2*		AAAAGTGTCTTGTGGGTGAGGG	GCGAAAATAGGTAGGCGGAG	Cs8g08330.1
	*VHA-c1*	V-type ATPase c subunit	CGCCCTTGTCTTCTCCTGTAT	GACTTGGCCTTGGGGTTAATC	Cs8g07570.1
	*VHA-c2*		TAACGCACAGCAGCCTAAGTTG	GATGAGAGGATGATTCCCACGA	orange1.1g031149m
	*VHA-c3*		GTATGGGACGGCGAAGAGTG	CAAGCGAGACCCGAAGACAA	Cs1g25080.1
	*VHA-c4*		GTACCGGAATTAACCCTAAGGC	CCAGCGGAGAGACCAGCAAG	Ciclev10002781m
	*VHA-c”*	V-type ATPase c” subunit	TCACCATATACCTTCTCCGCC	ATAATTGCAACAATGACCCCA	Cs4g20460.1
	*VHA-d*	V-type ATPase d subunit	CTTGGAGGCGATCGTGAGG	TACGACGATCATCTCGGGTG	orange1.1g018709m
	*VHA-e*	V-type ATPase e subunit	ATGGGGTTTTTGGTGACA	TCACTCCTCTTCACTCAG	Ciclev10010112m
	*VHP1*	V-type Ppase	CGAGCAGCAACAGCGACAAGA	CCACAGACCCCAGGAAAACGA	Ciclev10024946m
	*VHP2*		TGAGCCACAGAATCAGAGAGAGAA	GCACCAACAATCAAACCAATAAAC	Ciclev10007524m
	*VHP3*		CCCTGCACATACAACACAG	TGCTGACTCCTTTCCTTGCT	orange1.1g040141m
	*VHP4*		GTTGTGTCTTGGGGTGGTCTTT	GCCTTCAGCTCCATCTCGTATT	orange1.1g003697m
Actin	*Actin*		CCGACCGTATGAGCAAGGAAA	TTCCTGTGGACAATGGATGGA	Liu *et al*.^1^
