# Supplementary material for: Citrate Accumulation-Related Gene Expression and/or Enzyme Activity Analysis Combined With Metabolomics Provide a Novel Insight for an Orange Mutant
Source: Sci Rep. 2016 Jul 7;6:29343. doi: 10.1038/srep29343 (PMC4935991; doi:10.1038/srep29343)
Supplement: Supplementary Information [file srep29343-s1.pdf]

Supplementary information

Citrate accumulation-related gene expression and/or enzyme activity analysis combined with metabolomics provide a novel insight for an orange mutant

Authors：

Ling-Xia Guo#, Cai-Yun Shi#, Xiao Liu, Dong-Yuan Ning, Long-Fei Jing, Huan Yang, Yong-Zhong Liu*

Addresses:

Key Laboratory of Horticultural Plant Biology (Huazhong Agricultural University), Ministry of Education, Wuhan 430070, P.R.China

College of Horticulture & Forestry Sciences, Huazhong Agricultural University, Wuhan 430070, P.R.China

# LX Guo and CY Shi contribute equally to the paper.

Corresponding author: Yong-Zhong Liu

Tel: +86-27-87281897; Fax: +86–2787282010; e-mail address: liuyongzhong@mail.hzau.edu.cn

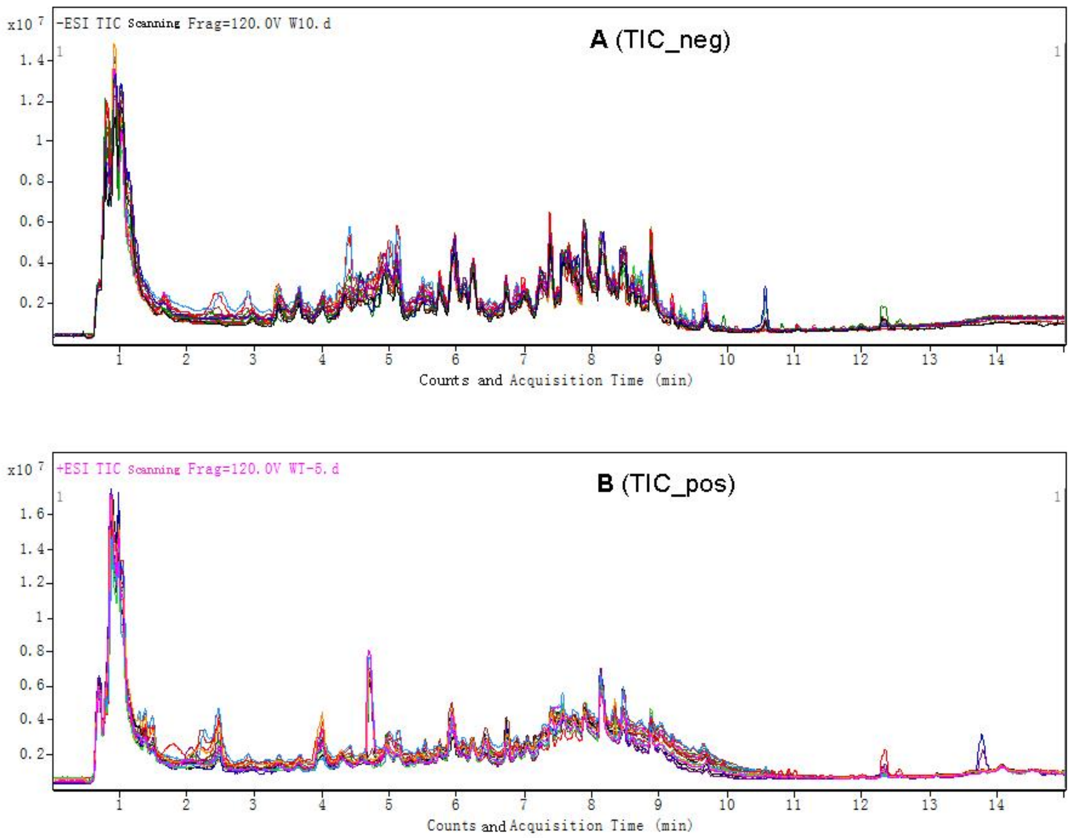

Fig. s1 Overlap of the total iron current (TIC) chromatographs of all samples under negative mode (A) and positive mode (B)

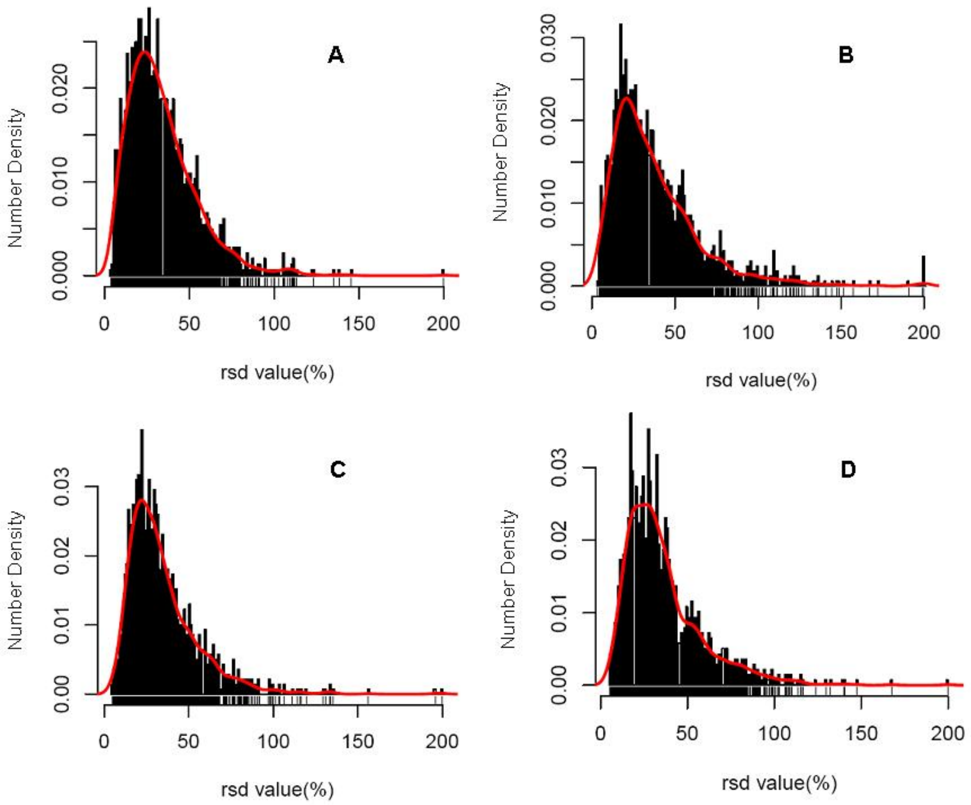

Fig. s2 RSD (relative standard deviation) frequency distribution. A refers to the RSD of group 'HAL' under positive mode. B refers to the RSD of group 'AL' under positive mode. C refers to the RSD of group 'HAL' under negative mode. D refers to the RSD of group 'AL' under negative mode

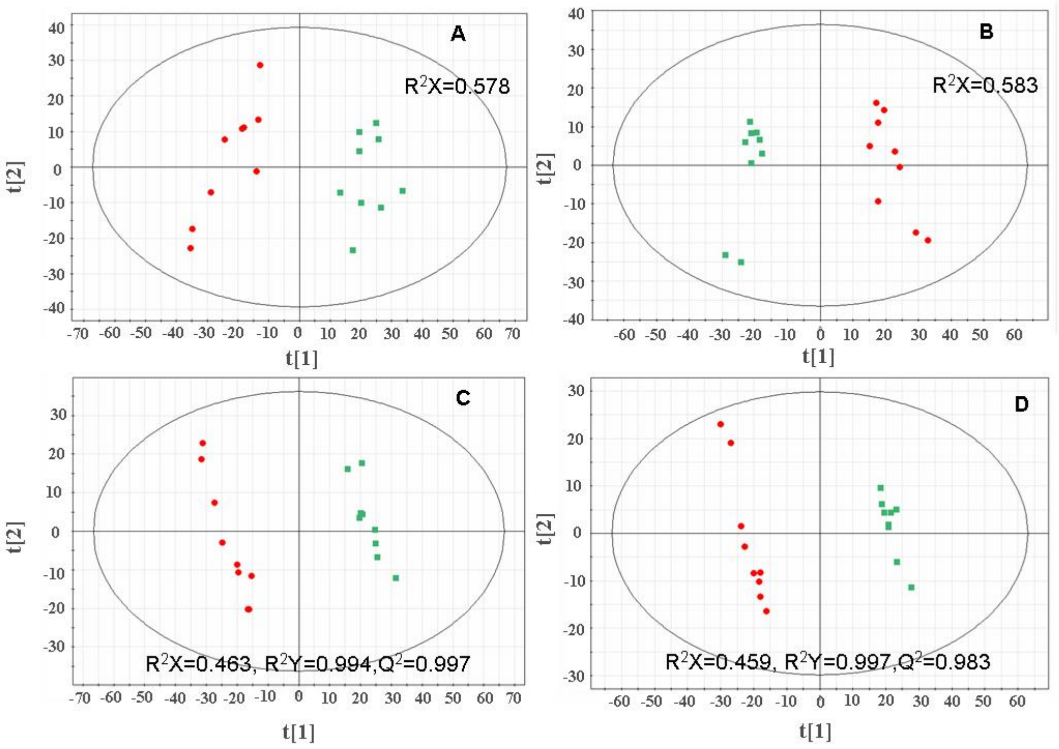

Fig. s3 The unsupervised principal component analysis (PCA) and partial least squares discriminant analysis (PLS-DA). A and C refer to the PCA and PLA-DA for positive mode, respectively. B and D refer to PCA and PCA-DA for negative mode, respectively. The red dots refer to the 'HAL' samples. The green squares refer to the 'AL' samples. R^2X refers to the sum of squares of all the X's explained by the extracted components. R^2Y refers to the PLS sum of squares of the entire Y's explained by the extracted components. Q^2(Cum) refers to the cumulative cross validated R2.

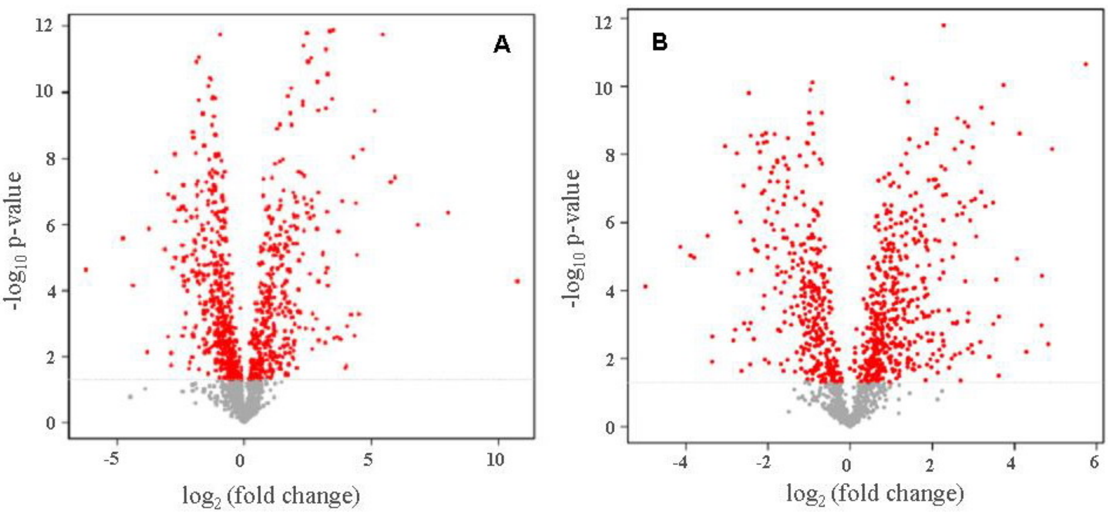

Fig. s4 Volcano plot for positive mode (A) and negative mode (B). Variables in red are significant at p-value <0.05.

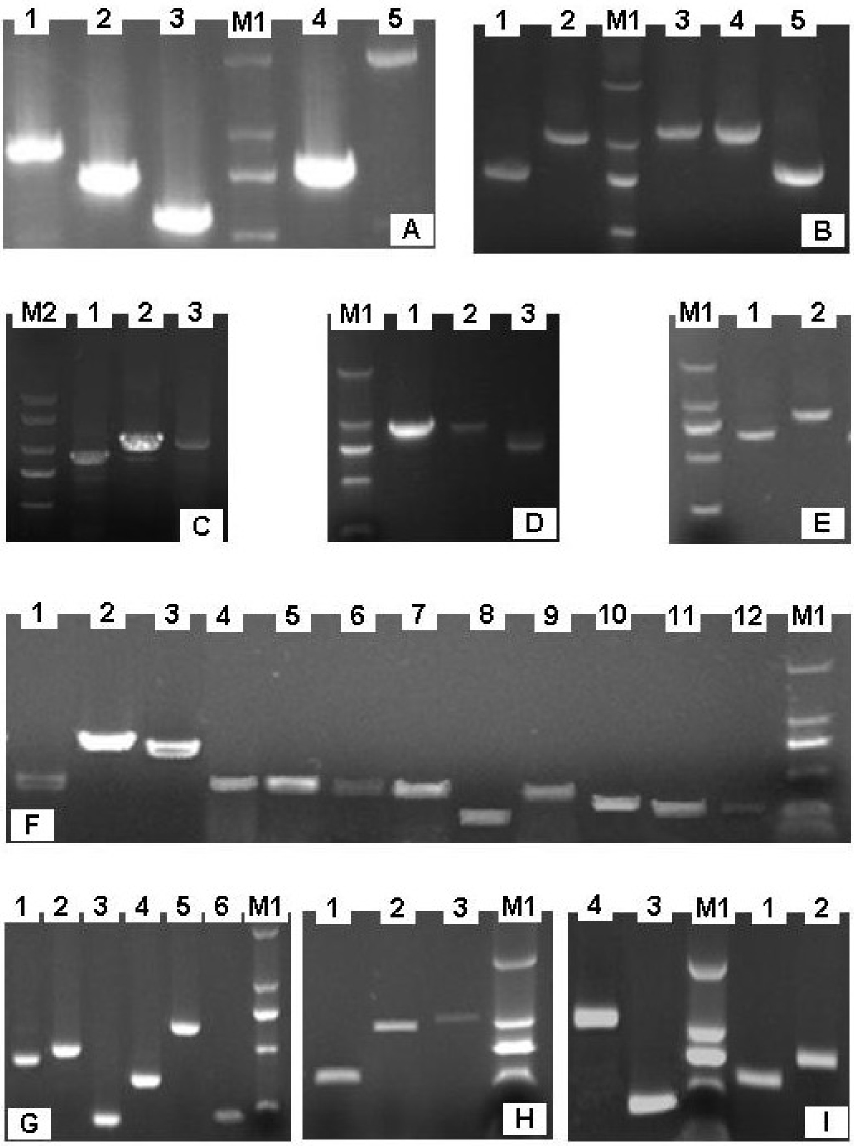

Fig. s5 PCR amplification of genes involved in citrate synthesis, transport, or utilization. A1, A2, A3, A4 and A5 refer to *PECP*1, *PEPC*2, *PECP*3, *PEPCK*1 and *PEPCK*2, respectively. B1, B2, B3, B4, and B5 refer to *CS*1, *CS*2, *GS*1, *GS*2, and *GS*3, respectively. C1, C2, and C3 refer to *NAD-IDH*1, *NAD-IDH*2, and *NAD-IDH*3, respectively. D1, D2, and D3 refer to *NADP-IDH*1, *NADP-IDH*2, and *NADP-IDH*3, respectively. E1 and E2 refer to *FBPase*1 and *FBPase*2, respectively. F1 to F12 refer to *VHA-af*, *VHA-A*, *VHA-B*, *VHA-C*, *VHA-D*, *VHA-E*1, *VHA-E*2, *VHA-F*1, *VHA-F*2, *VHA-G*, *VHA-H*1, and *VHA-H*2, respectively. G1 to G6 refer to *VHA-c*1to *VHA-c*4, *VHA-d* and *VHA*-e, respectively. H1 to H3 refer to *VHA-a*1, *VHA-a*2, and *VHA*-c'', respectively. I1 to I4 refer to *VHP*1 to *VHP*4, respectively. M1 and M2 refer to the DL 2000 marker and DL2000 ZM404 (ZOMANBIO, Beijing, China), respectively.

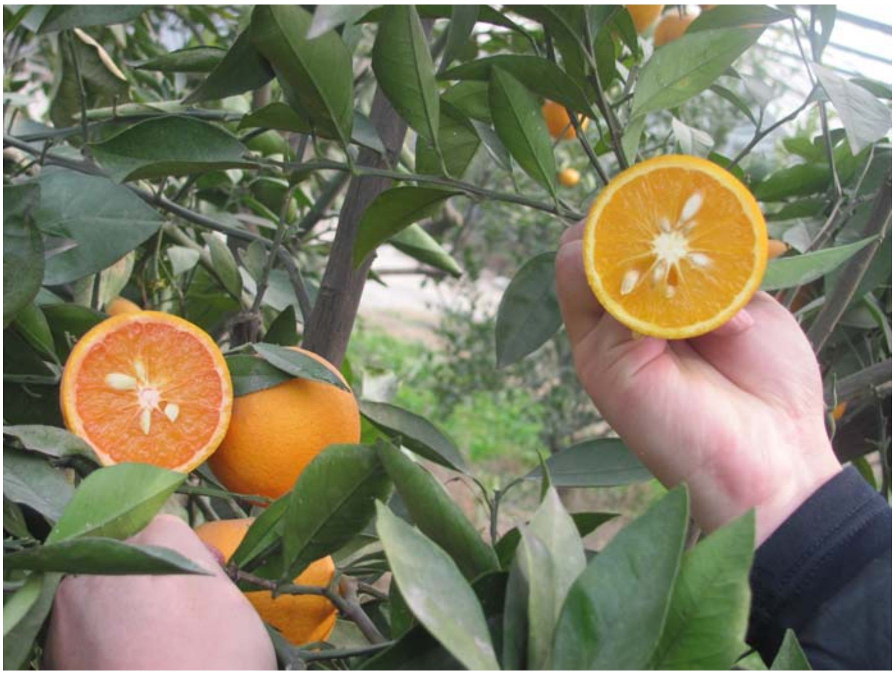

Fig. s6 ‘Hong Anliu’ and ‘Anliu’ grafted together on the same rootstock. The base and middle rootstocks are trifoliate orange and Satsuma mandarin, respectively. The left cut fruit is ‘Hong Anliu’ and the right cut fruit is ‘Anliu’.

Table s1 Significant difference in M/Z between 'HAL' (M) and 'AL' (W) under positive or negative mode

Positive mode					Negative mode				
M/Z	RT	VIP	p-value	Fold change(M/W)	M/Z	RT	VIP	P-value	Fold change(M/W)
595.1648859	5.94294814	1.66967	1.78879E-12	-0.942361935	100.0415049	1.40777129	1.47124	6.52951E-07	0.66072928
585.2158393	8.98078093	1.66888	1.63338E-12	2.471264291	104.0365584	0.77409569	1.21531	0.000484801	0.603294804
265.1533369	4.71429206	1.6659	1.78511E-12	5.44388615	111.0091768	1.02886032	1.56774	1.23296E-09	-0.930769689
567.2050195	9.67054751	1.66555	1.42837E-12	3.369511474	115.0044136	0.92323607	1.15438	0.001273197	0.427008508
583.1785637	9.67247525	1.6653	1.32392E-12	3.50439548	117.0202874	1.08010082	1.03167	0.006975775	2.674859042
580.2596543	8.97170087	1.6648	3.91902E-12	2.343730984	117.0205069	1.4037916	1.29385	0.000168266	1.144104968
562.2496763	9.67312481	1.65964	5.10792E-12	3.236585539	118.0520887	0.78598986	1.17764	0.000940175	0.533464725
236.07788	0.7609576	1.65598	1.61194E-10	3.453994289	120.9969982	5.3542159	1.33914	4.30245E-05	0.59573233
545.2230025	8.97771538	1.65561	9.45269E-12	2.633312337	128.0363262	1.04459364	1.48361	3.35384E-07	3.290869336
492.1771765	7.38934663	1.65535	8.92435E-12	-1.773703826	128.0363675	1.21993038	1.42509	3.50871E-06	2.171688562
149.0595194	4.72094502	1.65456	1.18858E-11	2.517268929	129.0204204	1.04457929	1.25736	0.000243697	0.561873087
967.3766417	7.39001093	1.65327	1.20404E-11	-1.875512348	129.0205202	1.2690318	1.06644	0.00387133	0.637160233
1000.993109	9.67611082	1.65054	2.90174E-11	3.291749951	133.0149573	0.92493701	1.2407	0.000337948	0.405497992
1084.325955	9.67611082	1.64868	4.89628E-11	2.88642522	135.031583	0.80972715	1.27956	0.000147382	0.81017202
455.1918352	7.39235476	1.6474	4.24647E-11	-1.290431167	142.0522528	1.41040584	1.46937	6.67733E-07	0.641980624
207.1378091	7.39240283	1.64735	3.7313E-11	-1.3489589	147.0311802	1.41012412	1.06108	0.004132983	0.312527245
163.1102763	7.38973291	1.64436	6.53656E-11	-1.409302621	153.0927456	7.39018894	1.47549	4.81201E-07	-0.802612083
177.0544209	4.7144162	1.6419	7.6079E-11	1.845638578	153.0928068	9.02706801	1.08002	0.003130287	0.562551906
729.2582685	9.19021863	1.64093	3.77465E-10	5.128292282	154.0633584	0.75223705	1.19654	0.000689124	1.049542459
427.2011419	4.60659331	1.64072	1.29156E-10	1.721970118	155.0000978	0.98130641	1.55792	3.34494E-09	-1.507665266
438.8957587	7.4965594	1.6394	2.45239E-10	2.309111018	155.0358456	2.44095831	1.29157	0.000117999	1.126791066
123.0802332	7.3924509	1.63877	1.55019E-10	-1.143811663	158.0473669	0.82092357	1.05812	0.004752403	0.582138703
149.0957624	7.39341346	1.63706	1.3668E-10	-1.218382807	158.97921	0.67567542	1.27234	0.000179381	0.329328935
173.0998195	7.7719487	1.6364	1.78625E-10	-1.784916725	163.0161227	8.83216441	1.56052	2.37699E-09	-2.046378848
495.1841116	7.39107239	1.63493	1.59185E-10	-1.252332243	172.0987858	6.12529966	1.3778	1.58033E-05	0.666315825
559.14477	8.34626948	1.63449	1.97277E-10	2.312333755	174.0783085	1.33695718	1.32584	5.85748E-05	1.155753975
565.1558847	7.21514362	1.62997	4.50231E-10	-1.628360375	174.9569376	0.66819347	1.08451	0.003856532	-0.416893591
483.1839565	7.31749063	1.62986	3.06902E-10	3.233952629	175.0255939	0.93330432	1.43234	4.39079E-06	0.923949239
393.1031647	9.67423282	1.62933	3.6195E-10	2.920705018	175.0621408	4.69766727	1.3082	8.64235E-05	1.828175117
473.2026993	7.39070612	1.62668	5.30577E-10	-1.189054206	183.1038027	7.98393418	1.26877	0.000212138	0.829065656
711.2552269	7.96620194	1.62658	9.84177E-10	1.866916884	186.0425165	1.41141832	1.48017	4.32855E-07	0.903881883
266.1021029	6.65537203	1.62643	4.31474E-10	1.813821316	187.0270105	1.40687875	1.41526	6.20289E-06	0.962259708
543.1320318	0.91340999	1.62369	1.27951E-09	1.299566306	187.0987954	8.33957251	1.36432	2.1483E-05	1.11386618
248.1120105	4.7247773	1.6209	9.59998E-10	1.401971362	190.0555094	3.72986689	1.09478	0.002995976	0.731520517
692.2189566	7.52355511	1.6188	2.3419E-09	-2.00024577	191.0213534	1.02860441	1.55251	4.80444E-09	-1.022598924

511.1574119	7.39240283	1.61876	9.90357E-10	-1.251194699	192.0833815	1.02781278	1.5602	2.55463E-09	-1.831680069
191.1060921	7.77744386	1.61592	1.68228E-09	-2.019386459	194.0830089	11.5863511	1.04319	0.004756682	0.123080158
256.0783608	7.39260358	1.61109	1.98056E-09	-1.129616217	196.0749057	6.09155082	1.40526	6.94816E-06	1.038732798
311.0762351	6.13136664	1.6081	1.27449E-08	1.394852368	203.0217284	0.94549425	1.21152	0.000507425	1.792134156
506.202716	6.35994941	1.60503	5.39292E-09	4.659098301	205.0367045	4.90160668	1.31351	7.2421E-05	1.051537824
221.1895053	9.69548463	1.60303	7.5662E-09	-2.723065018	205.0370727	1.75303813	1.19589	0.000721798	0.775125958
781.213979	4.95527096	1.60243	4.18274E-09	-1.573046108	211.0989768	6.09169406	1.42109	4.01882E-06	0.978729125
437.1828543	7.39187938	1.60113	7.58964E-09	-0.990539139	215.0348045	0.79993486	1.17209	0.001398276	0.610848454
159.0947315	6.1166256	1.60095	7.72973E-09	-1.113579432	218.1049902	2.99356153	1.34728	3.39847E-05	0.53502387
656.7300125	7.94956329	1.59991	1.07441E-08	1.530096888	218.1050188	2.67308091	1.39074	1.02312E-05	0.864842904
752.3333735	8.97589555	1.59838	7.23769E-09	-1.913344775	221.0683087	8.59790578	1.23506	0.000335788	-0.824751537
488.1914512	6.36053452	1.59755	9.38548E-09	4.289056035	221.1555411	12.591477	1.15739	0.001355531	0.157621358
113.0596215	7.3923233	1.59612	7.75618E-09	-1.086636421	223.1349087	7.39439249	1.17288	0.000956336	-0.587504707
249.0606992	7.38973291	1.59514	7.51656E-09	-1.019237812	226.0380701	2.87917682	1.23408	0.000359133	0.526020368
569.1707721	6.07642866	1.59428	8.17985E-09	-0.992602466	226.9211277	0.70942888	1.1963	0.000652728	-0.637101119
265.1433966	7.74625351	1.59306	8.66868E-09	-1.111239698	230.1049499	3.58851077	1.11179	0.002534724	0.797211019
483.2225222	8.78639035	1.59143	1.08032E-08	-1.378075944	232.8996702	0.73630889	1.52136	4.79224E-08	0.834074946
189.1269437	7.39399315	1.59116	1.19078E-08	-0.881856602	233.1197893	6.32914261	1.07409	0.00421614	0.859462889
657.2814808	5.96234999	1.5905	1.51904E-08	1.226892868	236.1059426	11.0537946	1.23147	0.000451241	0.61050018
595.9501703	5.939979	1.58816	2.92955E-08	-1.052162362	237.0093634	1.06026667	1.50063	1.62256E-07	1.473334307
161.0930421	6.73797564	1.58044	2.60059E-08	-0.762515003	237.009447	1.57126168	1.20952	0.000557129	0.677559068
298.1497383	3.20911904	1.58021	4.20882E-08	0.774945281	237.0637802	0.79266373	1.48899	3.03278E-07	0.839176533
229.122197	7.75074966	1.57996	2.18512E-08	-1.23493319	237.1514505	8.61489751	1.34711	3.66604E-05	0.86835607
448.2124747	8.3954139	1.57793	2.63276E-08	-3.471017836	239.1320666	8.61100126	1.25681	0.000242337	0.727650999
831.3245594	6.73732496	1.57779	3.05737E-08	-1.357304389	239.1669953	8.06222283	1.18714	0.000894323	0.63436144
843.2550655	8.21837063	1.57711	2.61777E-08	2.153721931	241.0116984	0.7260886	1.21447	0.000583437	-1.23237084
422.2022734	6.73712124	1.57699	3.57092E-08	-0.830185536	241.1096136	6.73803411	1.27641	0.000166339	-0.623052075
187.1110524	7.75160859	1.57673	2.72711E-08	-1.140688267	241.1466577	8.06248028	1.1023	0.00335398	1.22691591
247.1327592	7.75067581	1.57629	2.76956E-08	-1.345224262	242.177662	9.7445568	1.14048	0.001623137	0.611795882
346.1317568	5.24138314	1.57548	2.90997E-08	2.251986066	243.0643235	1.22161786	1.47065	6.03132E-07	2.60387246
601.240851	8.94338431	1.57527	3.89115E-08	1.103652244	246.9934337	3.57659337	1.24747	0.000378937	2.400953138
307.1766811	5.69424998	1.57402	3.84006E-08	5.926623996	246.993449	3.76748186	1.08846	0.004143868	2.28067661
225.1120078	5.24126955	1.57315	3.36741E-08	2.368299106	247.1202345	6.10010564	1.49962	1.50371E-07	2.337918234
352.1754396	7.76003611	1.5729	7.62711E-08	-1.143858253	248.9031357	0.72903806	1.35162	2.96269E-05	0.863883593
626.2337211	6.73389012	1.57192	5.28124E-08	-1.495844191	249.081218	5.76460041	1.33757	4.47904E-05	2.182901382
385.1505428	6.1082672	1.57177	3.70699E-08	-1.249328929	249.0998727	2.89815818	1.018	0.006654487	2.97781848
475.2189811	7.26400473	1.56802	4.91722E-08	-1.822572002	251.0795763	0.99522583	1.08496	0.003706474	4.821317535
463.1237329	7.79413571	1.56618	6.51359E-08	-1.193605483	251.1666497	9.69462228	1.4006	8.04923E-06	-1.468327127

427.2076005	3.35930056	1.56575	5.26048E-08	5.765641665	253.095136	1.20890514	1.06149	0.004002558	2.953110815
215.0158283	1.16277723	1.5632	6.3427E-08	-1.881544506	253.1095304	5.15348158	1.22853	0.000433964	0.523563644
438.9802723	1.15899155	1.56279	6.52012E-08	-2.393493196	254.0818808	2.99445512	1.36559	2.51465E-05	0.728353947
201.1294354	7.75067581	1.56116	6.86381E-08	-1.185497134	259.0242974	0.72979056	1.15603	0.001349218	0.516445145
424.1501773	6.73712673	1.56067	9.5689E-08	-1.265899032	260.0285214	1.39251324	1.21969	0.00049113	0.689969913
775.9236041	7.9547228	1.56051	9.36597E-08	1.557671514	261.075352	0.95781037	1.30228	9.79881E-05	1.295424333
692.4157734	7.2812864	1.55938	1.11152E-07	1.941929728	261.0897907	5.37674434	1.24776	0.000281512	0.423286081
510.1581573	5.98826065	1.55647	8.95808E-08	-1.380750141	262.0594031	0.82642088	1.09835	0.003071696	0.56651202
207.1013706	6.73707099	1.55606	1.39204E-07	-0.844190418	262.9270674	0.67598851	1.1211	0.001861023	0.318123486
165.0906616	5.79951497	1.55588	9.35164E-08	-1.146192506	263.1306367	9.02690587	1.04076	0.004884692	0.548413335
340.1606853	5.8467817	1.55581	1.25765E-07	-2.988696066	263.1306765	7.74594565	1.55269	4.53119E-09	-1.045972273
270.0950686	8.34772233	1.55556	9.94402E-08	-1.660088928	263.1306967	8.33642345	1.34674	3.40654E-05	-0.630602151
225.1120036	6.73733642	1.5536	1.52816E-07	-0.833777105	263.1422354	4.71989106	1.60078	2.24739E-11	5.736548559
496.2024761	7.63046316	1.55265	1.09309E-07	2.926969381	264.0894566	6.65531873	1.55312	4.65965E-09	1.876669962
880.0623947	8.75986411	1.55265	1.86573E-07	1.424561881	265.0953908	1.65345865	1.05865	0.004258641	0.898954793
119.0853999	7.39001093	1.55086	1.21753E-07	-1.248862209	267.1253957	8.82564443	1.21579	0.000585482	3.625934345
243.1225702	6.73841859	1.54925	1.56485E-07	-0.826711188	269.1046082	7.30406667	1.12918	0.002123336	0.795017274
213.1119454	5.15227338	1.54899	1.37448E-07	0.762310797	272.9607469	0.6758165	1.28275	0.000151057	0.508743644
727.2081823	5.3173441	1.54889	1.92986E-07	-1.425937472	274.0954869	1.44499006	1.58034	2.87122E-10	1.415117921
807.2365231	5.98699216	1.54884	1.34397E-07	-1.85558755	277.0945287	1.76260659	1.1023	0.002585263	0.663714827
352.1606589	6.10359716	1.54713	1.47473E-07	2.008866466	277.1310715	5.84412009	1.47495	5.05498E-07	-2.759965821
311.076357	4.54329409	1.54665	3.52955E-07	1.363398414	279.1106403	3.20578796	1.51247	8.24174E-08	0.986785518
222.0652619	6.73808155	1.54611	1.57366E-07	-0.843256456	279.1106811	2.83048486	1.45691	1.0596E-06	1.238042722
223.132564	6.57049306	1.54582	1.57043E-07	-0.862041832	281.136557	4.3152403	1.14487	0.00161758	-0.691693867
481.0905911	0.9615988	1.5457	1.62127E-07	2.359530357	281.1417998	8.61349015	1.30524	9.35078E-05	0.808106415
523.2125504	8.76786911	1.54569	1.57069E-07	-2.764945516	282.1219217	0.79008691	1.34295	3.66517E-05	0.879451367
165.0907552	6.73864971	1.54469	1.95402E-07	-0.816872545	283.1573842	8.06231298	1.1989	0.000738459	0.657835446
225.1119911	5.43316177	1.54316	1.82866E-07	2.644821234	284.1014928	0.77582653	1.19359	0.000696923	0.633595267
263.1278056	7.31805337	1.5418	1.96733E-07	3.851112894	287.0426258	4.30777358	1.2665	0.00018919	1.29025242
544.2051995	8.40502823	1.54077	2.298E-07	4.406631306	287.0428688	4.54580593	1.36834	2.22948E-05	0.661737118
440.2122254	8.47051296	1.54066	4.43048E-07	8.034100547	288.9891875	1.14383621	1.17467	0.000884465	-0.865390904
357.1156399	6.10398192	1.54052	2.11275E-07	2.071424733	289.1312807	6.10021233	1.49841	1.58844E-07	2.265430459
605.1851366	5.80200787	1.5404	2.15813E-07	-1.151000127	291.074308	4.04653925	1.15453	0.001911814	-0.652840296
203.1791366	9.70361114	1.53871	3.06408E-07	-2.435987765	291.0743371	4.32732125	1.01424	0.006555693	-1.29719118
535.178908	8.33905814	1.53839	2.25575E-07	-1.20329659	293.1258953	5.01272818	1.51676	5.86162E-08	0.694114069
594.235441	6.96748148	1.53788	5.1546E-07	1.949909344	293.1263877	4.40240536	1.11213	0.002366791	1.732257778
469.2430876	9.70268095	1.53782	3.54377E-07	-2.505805146	293.1264835	4.79406801	1.26706	0.000221785	0.648187505
655.1931736	8.04720335	1.53767	2.77139E-07	1.429996089	293.1778948	11.0536763	1.22256	0.000558233	0.616142606

346.1319777	5.43340829	1.53653	2.53327E-07	2.455766598	294.8954784	0.70056199	1.26405	0.000211046	-0.498567923
248.0810029	8.59983027	1.53602	2.5919E-07	-1.431734581	295.0697801	0.88204455	1.06592	0.003667581	0.847299536
451.2326191	9.70362068	1.53551	3.58615E-07	-2.608946768	295.0963241	1.47040702	1.09773	0.002518381	1.339792268
334.1648632	7.75433512	1.53461	4.08946E-07	-1.246151343	298.1171662	0.78852308	1.22989	0.000387584	0.544420722
375.0926	2.95876653	1.53357	2.9967E-07	-2.404539553	301.0737916	8.15901938	1.25116	0.000251721	0.404422834
773.2143732	5.16058067	1.53286	2.99908E-07	0.960617888	304.9157055	0.66805541	1.20229	0.000698911	-0.838717967
179.105779	6.73839305	1.53279	3.36636E-07	-0.699302958	308.9089538	0.66823455	1.20839	0.000581655	-0.772241178
498.2187552	8.4638415	1.53208	3.05143E-07	2.663225623	309.0494359	0.9357686	1.47442	1.06529E-06	0.695090363
137.0957858	6.73797564	1.5318	3.77626E-07	-0.793867997	309.1728152	11.2503729	1.00935	0.006821145	0.125105274
443.1881944	5.64417951	1.52908	3.58702E-07	0.862294911	311.1158686	7.30448409	1.11149	0.002766579	0.777951964
212.0079465	1.14970366	1.52907	3.52842E-07	-1.890286992	312.0980459	1.28945039	1.27121	0.000216016	1.230802761
523.2146911	8.37804551	1.52894	3.71185E-07	-2.300160688	315.0960319	0.80951509	1.23488	0.000336589	0.759695822
645.2756846	8.34878157	1.52796	4.75352E-07	-1.098901263	315.1831312	7.63653876	1.16947	0.00101149	-1.427535292
443.1317058	6.73733642	1.5278	4.2595E-07	-0.782583338	316.104082	5.15991488	1.30207	9.76991E-05	0.566308653
465.1523879	7.75518279	1.52597	4.13889E-07	-1.026679528	317.1988442	7.04424065	1.31148	7.57059E-05	-0.999121377
247.1327278	8.33824032	1.52585	4.08197E-07	-0.888449987	317.1989159	5.5502031	1.3406	3.95072E-05	-1.456538417
410.2020378	6.89262506	1.5254	4.14352E-07	3.267621754	319.0693987	2.75752122	1.16566	0.001118821	0.914214548
306.1610577	5.8592358	1.52484	9.24401E-07	1.075886925	321.1576672	7.7899249	1.18577	0.001008808	0.925744507
509.2358128	9.70446504	1.52403	6.05242E-07	-2.070069333	321.9067337	0.6508229	1.31402	8.04874E-05	-1.096794658
651.1091431	0.97615859	1.52393	4.47966E-07	2.48260922	323.1371276	3.79605241	1.31107	9.28011E-05	0.507520005
477.1761896	8.34217207	1.5205	5.2843E-07	-1.120995618	325.095296	5.13871446	1.16788	0.000989232	1.128430361
893.3035575	7.61597161	1.52	5.9377E-07	0.971166739	325.1371521	0.77414525	1.00425	0.007279171	0.684796607
871.248569	5.86925709	1.51971	8.97278E-07	-0.748107506	327.0743142	6.13281958	1.55818	3.55901E-09	1.446887453
589.2260635	5.79886307	1.51965	6.36551E-07	-1.029597851	327.0745752	4.55796383	1.41524	5.76001E-06	0.798001362
679.1843724	6.9182896	1.51913	5.74528E-07	2.09025125	328.0910651	1.65345865	1.05877	0.004499245	1.101843465
298.1496244	2.85132612	1.51847	1.21855E-06	1.42216782	329.0898539	2.95267872	1.52996	2.51772E-08	-2.191365049
410.2014669	6.49938697	1.5183	5.60143E-07	2.744093184	329.1546524	11.0537295	1.11327	0.002755591	0.52308241
587.0939394	1.14726765	1.51811	5.84777E-07	-1.860005964	331.1784719	7.95449321	1.40587	6.48053E-06	1.890533759
398.2007241	1.05024531	1.51748	5.96451E-07	1.980014118	334.1680899	8.56872403	1.05351	0.005521167	-0.561505827
529.3019521	4.71553416	1.51563	1.05704E-06	6.838106258	335.1369686	6.09097092	1.54968	5.88075E-09	1.675720204
133.0834795	4.68342669	1.51552	6.39136E-07	2.58209125	337.0806497	0.90816323	1.3004	0.000100828	0.992462333
541.1905809	6.40059079	1.51519	6.96807E-07	1.136913685	341.0918497	4.18930052	1.34444	3.53714E-05	0.911026091
344.090842	1.238777	1.51361	1.84527E-06	0.922908837	342.1073236	3.20272623	1.52446	3.73715E-08	0.947778033
587.2864429	8.25891518	1.51295	7.33028E-07	-0.794585933	342.1078147	2.82961766	1.30228	9.42337E-05	1.370711712
965.4588961	8.03612979	1.51258	8.19926E-07	1.593075649	347.0839168	3.99466612	1.28789	0.000125045	1.055229173
584.2551958	5.79972443	1.51203	7.75184E-07	-0.93289996	350.1634517	7.75055056	1.3827	1.51839E-05	-1.150711036
888.3479201	7.61173137	1.51196	7.36956E-07	1.064572483	351.0601288	0.93298785	1.3599	3.56578E-05	1.400181212
193.0326615	1.03187462	1.51089	7.94872E-07	-2.286046457	351.1321366	4.10623711	1.12372	0.001907566	2.233850969

407.0329537	1.15129103	1.50959	8.06355E-07	-2.712978242	354.1769241	7.05212495	1.35422	2.77149E-05	-0.840409989
322.5682942	7.21465941	1.50693	1.08143E-06	-1.651119167	355.0698703	3.77308399	1.36883	2.0571E-05	0.682731768
485.2384255	8.89320189	1.50679	9.05093E-07	-1.427132425	356.12167	4.40240536	1.0866	0.003216387	1.445056429
853.3767167	7.95069365	1.50528	1.36603E-06	1.441548807	356.12195	4.7831547	1.59268	5.79597E-11	1.03709608
706.2579682	8.01585629	1.50454	1.11458E-06	1.980299838	356.1220658	5.01353522	1.33689	4.23948E-05	0.567478419
430.162701	1.03730728	1.50312	1.33825E-06	2.069059228	356.1736343	11.0535561	1.22677	0.000490442	0.625147115
467.1888893	4.59864224	1.50132	1.2669E-06	-0.880789969	356.9942946	0.72704645	1.20307	0.000657853	0.855830443
949.2675189	8.0304143	1.50044	1.17576E-06	1.825781076	357.1203564	3.99843805	1.244	0.000286148	0.822548341
573.0980193	1.22394419	1.4986	1.24567E-06	-0.789656291	358.1336293	4.2904203	1.17481	0.00100179	-0.845736732
223.1288295	6.13085143	1.49793	1.27801E-06	-0.846452971	362.1477066	5.97348763	1.42811	3.10917E-06	1.552539615
321.1644255	6.07696052	1.49789	1.38367E-06	2.866657083	362.8482044	0.71658857	1.18769	0.000759406	-1.19012199
968.9142018	7.27849871	1.49757	1.66739E-06	1.666607085	363.068104	1.65574456	1.08026	0.003408015	0.793596127
147.1160671	8.39430518	1.49754	1.37014E-06	-3.739533241	365.1477936	4.70201203	1.18796	0.000749402	0.610665222
723.9307677	8.24363244	1.4955	2.1237E-06	1.407612942	366.122426	6.53405972	1.45106	1.34102E-06	0.614517509
270.0944513	7.98364288	1.49456	1.44754E-06	-1.25252986	368.1008375	2.81832316	1.03166	0.006720385	0.842417408
235.1689811	7.98009504	1.49437	1.45664E-06	-1.635444403	368.8306096	0.72859599	1.45107	1.35087E-06	0.991615693
478.2286178	7.31836551	1.49293	1.63207E-06	3.711697954	369.1581507	7.78322595	1.41669	5.1884E-06	-4.125115434
933.2856322	5.05631286	1.49224	1.57613E-06	2.357590038	371.173356	8.0841566	1.18113	0.000823501	1.421175693
444.2227344	7.74758603	1.49057	1.70711E-06	-1.056460908	371.1734108	8.26084026	1.11502	0.002017431	1.651463235
235.1689702	8.35464206	1.48776	2.02944E-06	-1.794358781	373.1893402	7.95552412	1.44876	1.46879E-06	1.588050115
483.1478602	6.09347931	1.48584	2.08955E-06	0.87951692	373.1894216	8.64394331	1.0226	0.007367956	2.021714315
807.4238036	7.2812864	1.48433	2.54951E-06	1.743746194	375.1328337	5.16676941	1.19348	0.000701751	-1.073685894
916.019559	7.80928071	1.48424	2.23119E-06	1.274744684	375.1685174	6.41954723	1.28862	0.000139425	0.550924535
197.1161696	6.73890491	1.48202	2.72475E-06	-0.721180673	376.0913131	3.49277136	1.12207	0.00183637	0.57582308
153.0901805	6.56628924	1.48201	2.28537E-06	-0.956762098	377.1842561	8.20388618	1.06395	0.00391222	1.454300908
449.1769782	7.75027146	1.48084	3.17009E-06	-0.712725257	379.1295749	5.79528992	1.47705	5.28291E-07	1.351478858
302.0398563	1.15146033	1.47979	2.84233E-06	-1.531887302	379.1634031	6.49777717	1.31295	8.32635E-05	1.574734356
527.2596215	3.97361237	1.4784	4.51495E-06	1.677322458	380.1586781	3.27657961	1.30699	0.000104421	0.74874859
393.0367333	0.96203515	1.47814	2.64247E-06	-4.760110789	381.0708549	0.91916323	1.30543	0.000120505	0.64697336
286.0922604	1.4660402	1.4781	2.92984E-06	1.792361101	381.0851866	6.6565532	1.43896	2.36432E-06	1.058853586
515.1193943	7.6816516	1.47742	2.68672E-06	1.014325703	381.1344289	6.87651164	1.04253	0.005152389	0.418396168
911.219135	5.9397854	1.4758	2.83302E-06	-0.848618791	382.1029038	3.22346997	1.07483	0.003831252	1.198269451
749.3386557	9.09272778	1.47425	3.81122E-06	1.928128928	383.1733606	7.1837815	1.3202	6.33734E-05	0.821547489
579.1712284	7.61769701	1.47321	3.36444E-06	-1.03139319	384.118484	5.84592166	1.4596	9.63751E-07	-2.663388784
131.0490022	7.15724287	1.47264	3.14463E-06	-1.950588587	385.0790389	4.50041121	1.18056	0.001228214	0.41531336
780.8626811	9.24743786	1.4712	5.92916E-06	1.15631981	385.079234	4.24655119	1.41062	6.88505E-06	1.169506589
226.5650991	4.97222791	1.47075	4.1734E-06	-0.96875264	385.1889244	8.39020136	1.39411	9.28571E-06	-3.879859908
504.2803511	9.70624958	1.4707	4.37195E-06	-2.426061722	386.941194	0.66775553	1.27012	0.000227107	1.12695419

501.2336101	7.97417866	1.46968	3.47858E-06	-1.282826083	387.1314498	4.5642155	1.35783	2.57601E-05	-2.399302605
147.0268383	1.02473661	1.46944	3.70467E-06	-1.92198911	389.0158895	0.92977636	1.21638	0.000713145	0.981026621
525.1209329	1.04508637	1.4678	3.96408E-06	1.639655978	389.1691713	6.15795211	1.27827	0.000195392	1.524152646
467.0846809	0.99942256	1.46672	3.84796E-06	2.072651265	389.183901	6.68108103	1.20015	0.000714698	1.326578614
449.7162389	8.40589531	1.46668	6.57947E-06	1.736042509	390.1536123	7.05245895	1.28667	0.000127548	-0.770376719
478.1915675	6.08794687	1.46659	3.86286E-06	0.796383855	391.1636017	6.88014934	1.51719	5.73044E-08	1.912902669
239.1276724	5.11212242	1.46645	3.955E-06	0.802972847	391.1636751	6.49434422	1.49242	2.3164E-07	2.951493613
979.2771918	7.99754755	1.46632	3.98708E-06	1.391051871	392.1739138	8.19446412	1.41042	5.64204E-06	1.064821749
918.9970196	7.71908157	1.46536	4.17293E-06	1.201728126	392.8964499	0.6800157	1.15604	0.001289778	0.518123795
702.7136164	7.36055957	1.46475	5.66088E-06	1.311754419	393.1428148	5.02605558	1.03471	0.006331364	4.288435233
574.1994899	3.96214176	1.46098	5.17962E-06	1.614913398	393.178543	5.61617574	1.1524	0.001300513	1.004869727
918.0785941	8.02215042	1.4607	4.8926E-06	1.041472611	393.178814	4.64925901	1.29027	0.000136958	1.85383083
342.2137987	4.58117116	1.46	4.75862E-06	-1.589425802	393.1791967	7.82738283	1.43253	3.26238E-06	2.071081742
969.87326	7.8074674	1.45982	5.03844E-06	1.226494219	394.9414251	0.72531892	1.17308	0.000958393	0.646231076
234.18109	0.72016141	1.45925	4.88513E-06	0.647804647	396.1169917	6.09604063	1.53127	2.25662E-08	2.220134365
576.2078286	8.3808468	1.45733	8.39323E-06	4.451482075	398.1687962	2.99361584	1.1845	0.000871838	0.758546531
605.2413904	5.63514747	1.45723	5.20015E-06	1.014029774	399.0955755	5.56889557	1.34607	4.04547E-05	1.031704628
411.1052669	4.96930272	1.45689	6.28536E-06	-1.274749995	399.0961515	4.89698394	1.29973	9.75321E-05	1.032072379
189.1593227	0.74139628	1.45544	6.13711E-06	0.964675465	399.1680815	6.74923241	1.59067	8.62442E-11	1.366104305
332.1239123	0.97444051	1.45528	5.73578E-06	0.75993284	399.1682614	5.11421593	1.46997	6.14084E-07	1.25884094
577.1921419	8.88928436	1.45412	5.73339E-06	-3.118871052	403.1626583	6.73790883	1.40823	6.77053E-06	-0.731467657
622.2341153	7.32739717	1.45308	6.56882E-06	1.320116959	403.1636775	5.80035629	1.53764	1.49597E-08	-1.770842835
399.2368763	8.52294245	1.45172	6.14692E-06	-1.941024966	405.0311979	1.19120134	1.30878	8.13488E-05	-0.848071058
727.2069741	5.74735855	1.45049	8.61054E-06	-0.842001696	405.1361174	7.77811326	1.49306	2.27071E-07	-1.867209025
267.1699298	3.85060208	1.44979	8.42104E-06	3.089646653	407.1135007	4.9709379	1.29725	0.000107725	-0.765862805
382.1709105	3.28837438	1.44924	6.63457E-06	0.651935674	407.1585455	6.05985118	1.1103	0.002815388	0.570206472
137.0829379	5.81473948	1.44845	7.20195E-06	-1.179923593	407.1589452	6.46928166	1.23117	0.000459862	2.882226821
658.2553378	6.91571541	1.44752	7.01546E-06	3.102317597	408.1536526	6.87310531	1.31571	7.39073E-05	0.756486167
571.2449389	5.06610523	1.44534	8.87606E-06	1.435316514	411.2052483	8.60256138	1.32378	5.75278E-05	-0.978108364
129.01566	1.02577294	1.44469	8.23674E-06	-2.030918501	413.1480244	7.74557517	1.50797	9.4728E-08	2.081838817
663.2226631	6.92384192	1.44454	7.78229E-06	2.31339271	414.1276372	4.09459607	1.12573	0.001862979	2.021363219
513.1964499	8.33731707	1.44324	8.54506E-06	-0.587595362	414.2230872	8.66023678	1.34944	3.12235E-05	-2.715253177
609.1816329	8.71943823	1.44133	8.7551E-06	1.24011648	415.1633232	6.09140757	1.38065	1.37423E-05	0.823071807
1030.427828	7.80790061	1.44098	8.78412E-06	1.160298787	415.1639564	7.77998568	1.5526	5.78583E-09	-3.037952755
479.1887706	8.59983491	1.43927	8.94933E-06	-0.94076531	416.1679304	7.76642944	1.47634	5.4852E-07	2.733157992
675.8457728	7.47233711	1.43913	1.02502E-05	-2.706683259	417.0853014	8.89953177	1.0573	0.004252415	1.150177743
533.8780915	4.95557629	1.43912	3.57034E-05	1.008928008	417.0853132	9.19434891	1.05051	0.004519361	1.257708857
724.3835494	8.03725979	1.43852	1.05024E-05	1.439973519	417.1430901	4.96970812	1.33957	4.19083E-05	-0.646927416

209.1531835	8.58973064	1.43785	9.56679E-06	-1.151870242	417.1728838	7.05538835	1.11646	0.002024052	-1.111935482
450.2339237	6.71319308	1.43782	1.08463E-05	-0.892701966	418.1015405	7.30448409	1.14527	0.001728096	0.776419965
1037.344504	7.98526542	1.43741	9.44737E-06	1.530069796	418.8964703	0.66775553	1.05658	0.005479842	-0.531835605
700.2278714	5.74804241	1.43684	9.5525E-06	-2.261124982	419.1505399	7.18269255	1.36367	2.23047E-05	1.080136934
390.1760951	4.97149024	1.43564	1.03466E-05	-0.88751648	419.1583581	5.15651548	1.47253	5.50791E-07	0.887005174
536.8964606	4.95564021	1.43558	3.50352E-05	1.010727101	419.1948432	7.94988391	1.42222	3.84769E-06	1.843126325
211.5596052	4.56624199	1.43419	1.0432E-05	-2.371762034	421.0053975	1.14978377	1.43149	2.77989E-06	-0.957070382
525.2309687	8.55121924	1.42996	1.17414E-05	-1.87193816	421.1659241	5.99431514	1.27934	0.000166801	-0.740822712
233.0774368	7.74740682	1.42928	1.53765E-05	-1.073109656	421.1739477	7.4727524	1.30888	8.53713E-05	-1.822026546
536.2333713	6.40353431	1.42881	1.24559E-05	2.156606933	421.1742922	7.7994719	1.14475	0.001788597	1.881730175
588.2664292	7.39565692	1.42844	1.21219E-05	-0.835021363	422.9053791	0.67771749	1.31621	8.59688E-05	-0.761765568
314.1060747	5.75005013	1.42615	1.29262E-05	-2.381658308	423.1887416	5.73974361	1.43455	2.47988E-06	1.742076907
241.1518447	1.04518963	1.42613	1.54095E-05	0.557589339	424.8570469	0.7004518	1.3003	0.000117764	-0.773613531
898.42334	8.41125524	1.42586	1.36867E-05	0.705128454	425.1845038	7.74736291	1.58691	1.26707E-10	-0.965240328
421.1851136	8.59381843	1.42431	1.74309E-05	-1.552847106	427.1987864	7.38940686	1.57437	5.89515E-10	-0.987549738
387.199853	5.99570046	1.42414	1.371E-05	-1.051561772	427.1996556	6.90328926	1.52682	3.41434E-08	-1.910579614
193.0857655	4.97203751	1.42384	1.47999E-05	-0.713174314	427.9133203	7.39143602	1.50418	1.27737E-07	-1.154263348
767.2727277	4.96952892	1.42346	1.68214E-05	-1.786082077	429.2152355	7.9969548	1.32768	5.93775E-05	-0.634237288
407.1675876	7.18988441	1.42338	1.45688E-05	0.688199865	430.1836932	7.18256309	1.22754	0.000395238	2.153166977
325.1282024	8.48152011	1.42325	1.53766E-05	0.675258798	430.8149531	0.70748651	1.09323	0.002679248	-0.888349271
457.2073425	8.60463872	1.42276	1.44816E-05	-1.29078705	431.1008758	7.4573218	1.55474	4.49539E-09	-2.100283137
709.1993999	7.74252118	1.42269	2.40156E-05	-0.854952962	431.1235566	5.39578699	1.38725	1.14429E-05	0.563685845
392.1914748	5.15174883	1.42237	1.72083E-05	0.610000085	431.1945796	5.98638276	1.4321	2.7887E-06	-0.919701803
227.093828	7.26338944	1.42101	1.50619E-05	-1.100940821	432.1540569	7.77551093	1.45838	1.13636E-06	-1.904523957
191.142706	8.59983491	1.4207	1.5298E-05	-1.061912136	433.1742764	7.63551758	1.49028	2.61085E-07	3.478970117
220.1179724	2.68856518	1.42029	1.51665E-05	0.747911188	433.1744262	7.98939159	1.08714	0.003530591	1.000113407
427.1575386	6.73881047	1.42018	1.52595E-05	-0.67365181	434.873486	0.66786138	1.21865	0.000498264	-1.084382536
393.199093	7.63004865	1.4183	1.64758E-05	1.511607796	435.1458602	5.11874206	1.19676	0.000630049	0.844940716
603.072096	1.23748671	1.41779	1.67676E-05	-1.405960083	435.1899497	8.45521638	1.00167	0.007771213	2.549249782
265.1436301	4.33279259	1.41662	1.7388E-05	-0.902877067	435.960594	1.17601834	1.51021	8.35876E-08	-2.584691078
207.1378109	5.98910705	1.41649	1.72437E-05	-1.172506665	437.2203092	9.56193864	1.37825	1.7023E-05	-0.917538948
439.1974423	8.59983491	1.41555	1.74697E-05	-1.072119429	437.2204947	8.79102128	1.43195	2.77259E-06	-0.935750978
307.0759553	0.97444051	1.41344	1.81888E-05	1.452302145	438.0919126	0.93005633	1.48584	5.13839E-07	1.290007462
625.1768529	6.3926023	1.41144	3.65073E-05	-0.73573187	439.2358621	8.92371857	1.37125	1.77678E-05	-1.26401883
693.2009616	8.70791736	1.41103	2.11683E-05	3.269227991	440.8375112	0.70056199	1.32729	5.37467E-05	-1.163487021
819.4217773	7.36055957	1.41053	2.15525E-05	1.318534541	441.1788222	5.65529376	1.17723	0.000849623	1.08825109
1099.049079	7.80864655	1.41	2.08754E-05	1.117665438	441.2519026	9.70315528	1.43074	3.30102E-06	-2.345697767
420.2585963	5.66241864	1.40892	2.07763E-05	-2.807083552	442.1344132	6.46045338	1.0865	0.002995989	2.263809142

549.1582569	8.40305224	1.40826	2.35943E-05	1.724227941	443.1945639	6.57256351	1.47649	4.67218E-07	-0.842768579
569.3202185	7.86808709	1.40819	2.56117E-05	1.795526658	443.1946709	5.74253188	1.40576	6.90755E-06	-1.079718562
1075.307786	6.23401388	1.40717	2.94036E-05	-0.613462671	443.1947988	4.59998537	1.3256	5.5343E-05	-0.549866406
286.0923792	1.89386522	1.40629	2.40259E-05	1.783606431	445.1590156	1.6796475	1.41933	4.26607E-06	1.051478486
867.8764665	7.8082616	1.40462	2.32254E-05	1.072409921	445.1740052	5.11141874	1.44522	1.68424E-06	0.703502066
557.1843779	4.46348503	1.40439	2.79093E-05	-1.09383844	445.2469226	8.60550597	1.35251	2.89602E-05	-1.007129702
555.0906548	1.0080398	1.40296	2.37364E-05	-6.22496152	446.1692414	7.1825559	1.33802	4.12922E-05	0.904174543
111.005354	1.02829932	1.40281	2.6522E-05	-2.033401877	446.8210644	0.7084668	1.14171	0.001614769	-0.824578525
139.0006109	1.0290774	1.40251	2.72551E-05	-1.752184925	447.0985904	6.67482622	1.37859	2.19269E-05	-0.847925996
279.170129	6.94275242	1.40229	3.54768E-05	-1.827994327	447.1895862	4.1795071	1.4125	5.24063E-06	-0.617751248
289.6684705	5.19334598	1.40118	2.54577E-05	-1.124686014	447.1897439	4.53346206	1.37278	1.87343E-05	-0.709452323
163.1110901	5.99073765	1.40075	2.59072E-05	-1.114754553	448.1849143	5.99039986	1.29988	0.0001081	-0.930965759
356.1890527	7.04975088	1.40025	2.54024E-05	-1.074163754	449.1115721	6.05510031	1.23518	0.000465236	-0.761782146
334.0451905	5.10179749	1.39966	2.90381E-05	-1.128790021	449.1974278	7.26396908	1.26198	0.000206422	-0.825336422
807.3414739	9.03502954	1.3983	2.73306E-05	1.791182419	450.2001158	6.87194221	1.26262	0.000222047	-1.130434875
161.1318567	7.39390481	1.39824	4.02054E-05	-0.620326145	451.1492444	5.88783194	1.10848	0.002690965	0.513134657
613.1539403	0.98270355	1.39742	3.14587E-05	1.147052789	451.2094363	7.77293984	1.2339	0.000437519	1.753571662
619.1800353	7.91603955	1.39673	2.77524E-05	-0.913922002	453.2146534	7.80112383	1.35258	2.88552E-05	-1.562478211
475.1429574	6.47431894	1.39662	3.13022E-05	2.582860858	454.0196111	11.0525421	1.06081	0.005123485	0.965297641
523.2138833	9.69445452	1.39557	3.61167E-05	-1.438275668	454.158912	6.88227829	1.49547	2.02883E-07	3.042987694
191.1426922	8.17942499	1.39538	2.98852E-05	-1.64941348	454.1591837	6.49403753	1.5489	6.16896E-09	2.988625816
157.0106192	1.03151022	1.39533	3.13237E-05	-1.887415115	455.2310131	8.35178389	1.50281	1.40529E-07	-2.140336557
447.1263896	8.31298878	1.39502	2.89685E-05	3.221645677	455.231511	7.98288149	1.43046	2.86576E-06	-1.009301346
664.2254169	6.95027205	1.39493	2.89646E-05	-1.902529585	457.1230142	1.37231287	1.33807	4.73213E-05	3.554886994
211.1685957	8.65611568	1.39311	3.0273E-05	-1.758664836	457.2468667	8.5756103	1.17492	0.000900297	-2.418338718
870.671253	7.72143111	1.39277	3.14474E-05	0.939400736	458.2493072	8.56626109	1.13503	0.001580896	-1.692061456
367.1369088	6.14000668	1.39225	3.09142E-05	-0.4532551	459.2256009	7.26573983	1.26276	0.000203751	-0.659527976
427.2056116	1.07057986	1.39123	5.30577E-05	10.75179243	461.0999881	1.02717626	1.46234	8.50737E-07	2.265067872
409.183445	5.99059466	1.39109	3.4242E-05	-0.928836525	461.1600398	7.75009455	1.56681	1.25048E-09	-0.995079714
242.0812812	4.32824743	1.39085	3.20133E-05	-1.136008965	462.1640429	9.32283769	1.07769	0.003236615	1.575899355
688.3183406	7.39118284	1.39077	3.20488E-05	-0.937663575	462.164303	5.11728622	1.30166	9.60395E-05	0.86550344
230.9856886	1.0119501	1.39017	3.46289E-05	-1.596519268	464.1799784	5.76462248	1.15435	0.001250737	-0.577565514
178.5980503	7.05128457	1.38935	3.31481E-05	-1.406679855	464.180454	4.17513596	1.43188	2.74482E-06	-0.880594075
701.9182445	6.87775762	1.38768	3.66458E-05	-2.406686135	465.1426611	7.18228894	1.29999	0.000100604	-0.875279419
210.0599546	1.15529504	1.3872	3.83869E-05	-1.917330851	465.2003242	6.87249164	1.00174	0.007445172	1.212507474
161.0594543	4.97231702	1.38711	3.6114E-05	-0.65635065	466.1592971	6.73942428	1.363	2.39376E-05	-0.495554316
467.1902469	4.34094854	1.38473	3.89006E-05	-0.903992267	467.1221078	8.06189344	1.1204	0.002770608	-0.728227745
283.1623799	1.04777881	1.38138	5.3525E-05	2.928720855	467.1221371	8.58419154	1.18699	0.000980887	-1.148839344

137.0958431	8.59866534	1.38075	4.06486E-05	-0.954317949	467.1948512	8.33877842	1.39846	8.1278E-06	-0.786168971
123.48535	0.72109256	1.37862	4.30363E-05	-0.704889285	471.189955	7.3896366	1.5599	2.45295E-09	-0.896180232
837.7557407	7.42553066	1.37727	4.40323E-05	1.067171895	471.1902438	7.74661804	1.59046	7.71612E-11	-0.913305252
395.1313846	4.97193623	1.37703	6.08734E-05	-1.109135113	472.1608326	3.93149836	1.03024	0.006153083	0.350260137
173.4312201	0.70510175	1.37493	4.74918E-05	-1.599746482	473.1318399	7.87648274	1.516	9.23199E-08	-1.509672998
648.1115407	5.10179749	1.37464	4.80176E-05	-2.19239595	475.2211647	8.69040576	1.56543	1.50504E-09	2.871898583
663.2568661	7.5370993	1.37192	5.26651E-05	1.661825983	475.2545637	8.05719181	1.42193	3.95179E-06	-1.404699943
369.1522668	6.88464189	1.3712	5.92385E-05	0.658579574	476.2163848	7.26389167	1.19033	0.000707932	-0.679658441
699.8491718	6.10438982	1.37114	5.31486E-05	-1.837794384	477.1495572	0.81887995	1.04071	0.005693587	0.682260982
329.1593811	6.87169901	1.36992	6.73757E-05	0.808138825	477.1642068	7.6602657	1.39242	1.17331E-05	4.06534889
618.2593206	1.04863058	1.36946	7.10394E-05	3.294469138	479.1714386	4.32769481	1.24813	0.000271828	-0.733919743
131.0490117	4.56552619	1.36925	5.30514E-05	-2.351098195	480.1746614	7.94788727	1.46187	9.2076E-07	1.612854297
251.1278404	7.05671427	1.36827	5.72758E-05	-0.8952047	480.1760845	8.65215565	1.10336	0.00296612	1.982027802
337.053114	5.10179749	1.36741	6.49754E-05	-0.893422624	483.2253728	8.78507617	1.41311	5.82228E-06	-0.940346933
672.3218779	8.59844821	1.3669	5.57977E-05	-1.106586248	485.2052305	6.0505598	1.12952	0.00173238	-0.449153545
285.0876678	6.03758453	1.36568	5.83866E-05	-0.581557592	487.180003	0.80694232	1.07202	0.003662385	1.092764036
423.0898927	5.55990792	1.36543	6.71671E-05	0.743535154	488.1392105	6.73808453	1.16287	0.001314704	-0.737758031
227.1640523	5.92301415	1.36308	6.33426E-05	-1.750096488	488.1673539	0.82070102	1.28302	0.000152017	-1.344103584
683.215874	7.41125438	1.3628	6.11595E-05	1.595890419	488.1801131	7.74830532	1.48766	2.72525E-07	-0.706248947
744.7905801	7.42394068	1.36271	6.20627E-05	1.125088044	488.8951534	0.67728182	1.28488	0.000134217	0.686388536
552.2285204	4.46688917	1.3619	6.39361E-05	-1.052821377	489.2006756	4.32408685	1.37817	1.51032E-05	-0.964829196
525.1221029	1.34240966	1.36122	7.71596E-05	1.213356148	490.1244427	7.15639811	1.43191	2.8208E-06	-1.613616035
644.1250594	4.9549583	1.35987	0.000174761	0.910176524	490.1804829	4.56615099	1.05251	0.004281053	-0.853673284
152.1055922	1.63395222	1.35932	9.78984E-05	-1.258011822	491.1711447	8.59242195	1.30988	7.85899E-05	-0.951685536
189.1269695	5.99437127	1.35851	6.89018E-05	-0.678891185	491.1810203	5.33700718	1.24066	0.00037592	1.313895614
372.1874329	5.06992143	1.35814	6.73576E-05	-1.073636165	492.1750117	8.39080744	1.38918	1.07394E-05	-3.794325927
397.2217629	8.19123786	1.3581	7.06924E-05	-4.37960907	492.885287	0.71645005	1.02125	0.006655892	0.331221548
248.0737245	5.01630378	1.35796	8.15578E-05	0.594270508	493.158895	6.73607015	1.41314	6.22861E-06	-0.878362751
581.0612554	7.27812991	1.35746	0.000117117	1.600570569	494.0967413	7.4483303	1.50587	1.16595E-07	-2.087236531
817.2004674	1.05633145	1.35636	9.5012E-05	2.143450237	495.1742066	5.83821365	1.18732	0.000848911	2.604897932
350.090486	3.86423123	1.35625	7.24962E-05	0.835934254	497.1818051	6.37299429	1.33809	4.1199E-05	-1.124402943
599.1977095	7.59646343	1.3561	7.04266E-05	-1.28355481	498.1575215	6.8803947	1.12809	0.001759645	-1.031906759
253.179744	7.26756215	1.35603	7.2558E-05	-0.919580911	499.1333636	0.95475144	1.39594	1.04045E-05	2.505820029
350.0908314	4.22578575	1.35569	7.19905E-05	0.822507772	499.2179063	8.34973979	1.34916	3.40194E-05	-1.766844773
682.2353681	7.51150802	1.35538	7.38474E-05	1.496417044	499.2207496	9.67241361	1.56696	1.23638E-09	3.474053212
163.0749716	4.56681369	1.35436	7.36177E-05	-2.370362816	500.9197652	0.66797613	1.2165	0.000677391	1.62330608
415.2332646	7.2659095	1.35347	7.71273E-05	-0.973790615	501.1070025	7.68922278	1.51089	8.17026E-08	2.148583306
1005.096411	7.42540809	1.35059	7.96953E-05	0.993735816	501.2361566	7.99154749	1.26777	0.000184658	-0.882062537

123.0810114	5.98780179	1.34882	8.7585E-05	-0.87269715	504.1750432	6.69940382	1.25562	0.000488044	0.826130655
235.1690902	7.26896963	1.34858	8.56928E-05	-0.926233192	504.1898373	6.36090245	1.55114	7.00554E-09	4.917370314
397.2221056	7.26681254	1.34798	8.69707E-05	-0.905230587	505.1013025	6.98501042	1.37258	3.3704E-05	-0.689003074
337.5737354	5.94219477	1.3478	8.97542E-05	-0.470126779	506.1900948	4.60086209	1.09171	0.002697117	-0.45628478
804.2884647	7.42551706	1.3436	9.17869E-05	1.003744117	506.1902275	4.32425552	1.31714	6.90667E-05	-0.8830329
149.095722	5.99041331	1.34328	9.57808E-05	-1.037772684	506.8806486	0.68130559	1.25025	0.000256476	1.055281571
379.186145	7.56237281	1.34319	0.000122527	-0.634858168	508.1115804	9.95031098	1.36676	2.06652E-05	0.455444087
338.1784194	7.04953288	1.34237	9.38588E-05	-0.948962538	511.1841219	8.33591718	1.31162	7.89657E-05	-0.62637943
913.8187173	7.42414036	1.34082	9.70252E-05	0.98573568	513.1981745	7.75032256	1.19967	0.00064681	-0.890145047
136.9353828	0.72498771	1.34034	0.000114328	-0.805299976	513.2280032	8.65009151	1.07222	0.003648116	0.840198278
361.1750653	7.5625716	1.33997	0.000120833	-0.630010314	515.1282507	1.00765555	1.43455	2.47807E-06	-3.463652586
718.2292156	7.42553066	1.33858	0.000103031	1.050875623	515.1794504	6.19029161	1.2421	0.000398918	0.933661695
181.0494654	4.19566948	1.33849	0.000106209	0.915793618	516.1679818	4.1652815	1.13669	0.00206182	1.650912055
360.1639277	4.57025604	1.33785	0.000103802	-2.236750469	516.8989768	0.66775553	1.39016	1.04087E-05	0.591506916
667.1890691	7.23619859	1.33761	0.000106062	-1.217683385	517.2316003	8.97958836	1.5772	4.18219E-10	3.201808857
1042.849617	9.60299674	1.33733	0.000236146	1.716277375	518.1906171	8.59395708	1.32505	5.61193E-05	-0.945496428
203.1791296	8.95920723	1.33492	0.000109531	-1.305480248	519.1171967	8.35482969	1.04675	0.007571725	1.227916464
246.1812273	0.74403885	1.33323	0.000113055	0.673425545	519.1753098	6.81290839	1.04589	0.005568605	-0.999504553
699.193366	7.41229538	1.33228	0.000116168	1.488649939	519.2465536	7.86909006	1.01344	0.006726264	-1.133522565
455.114548	0.77764686	1.3322	0.000120316	0.755336768	519.2467826	8.06309074	1.32459	5.66155E-05	-1.598163045
171.993117	0.68936603	1.33101	0.000141795	-0.79443344	521.1109835	10.627237	1.03837	0.00510539	2.872765002
553.1888811	6.05006266	1.32966	0.000121519	-0.789812347	522.1493476	6.08764796	1.39112	1.01204E-05	0.996000723
530.217991	5.89912159	1.32749	0.000141767	-1.239026086	523.1149493	4.94747901	1.16754	0.001394652	0.975299928
321.0973208	7.30994345	1.32674	0.000174834	0.826645931	524.1101993	7.77448031	1.18828	0.000776019	-0.994802419
1033.741329	7.72028792	1.32578	0.000133458	0.992889094	524.2007098	6.37954746	1.25077	0.000253382	-1.003614723
963.2767345	8.21856113	1.32415	0.000148435	1.640558798	528.1597149	1.40687875	1.04748	0.00534792	0.779494838
614.1406839	5.94058137	1.32183	0.000147353	-0.455621005	533.0959802	5.71637294	1.37087	2.4908E-05	-1.163943218
319.0814048	6.53800928	1.32136	0.000192489	-0.547510905	534.1855849	7.39453192	1.53414	1.85233E-08	-0.682993095
217.1566983	7.05977022	1.32093	0.000143758	-0.814229219	536.2007471	7.99084115	1.29567	0.00010991	-0.476012564
828.3835227	7.06688597	1.32018	0.000162351	-1.060607343	536.2049862	7.26545269	1.53242	2.36046E-08	-1.861306337
948.424814	7.05219966	1.31955	0.000153538	-1.756179927	537.1474286	3.7534711	1.45495	1.20277E-06	-1.292369937
230.4232413	0.7048809	1.31574	0.000176742	-1.182607462	537.1640397	8.52707947	1.12443	0.003042814	1.908167897
542.2527921	7.50531245	1.31483	0.000174703	-1.045632632	539.2507053	8.82302199	1.07277	0.003419939	0.753906045
973.8040665	7.726825	1.31447	0.000163653	1.199083955	540.2410852	7.50225915	1.13219	0.001686023	-0.9732717
849.2835551	7.65368106	1.31328	0.000166748	0.905895221	541.1635921	4.6005817	1.03174	0.005392512	-0.322722388
561.217722	8.23756159	1.31315	0.000166664	0.860899441	543.2102487	9.67511313	1.55367	4.30202E-09	2.720086379
773.3929612	7.42686973	1.31302	0.000170855	1.063648505	544.2059417	8.78930007	1.46586	8.52276E-07	-0.871744359
291.0419891	0.99326688	1.31048	0.000269866	2.003439521	544.7968501	0.70266276	1.07026	0.00349587	-0.694038542

255.9439605	0.67547928	1.30996	0.000249782	-0.866887299	545.1332864	8.28175154	1.55985	2.51221E-09	2.089906391
214.9175769	0.67532807	1.30925	0.000237151	-1.009020478	546.2211224	8.89680232	1.32732	5.44874E-05	-1.13933811
344.0890302	1.04050942	1.30843	0.000237597	0.710062627	547.111698	6.82351159	1.26512	0.000455421	-0.507256009
695.2175963	9.11457812	1.30798	0.000230366	1.777823331	548.2367931	9.70192076	1.39322	1.07557E-05	-2.000487784
678.2602772	7.41235167	1.30763	0.000184657	1.587727477	548.8532283	0.66827045	1.14372	0.001837654	-0.813352542
143.9961655	0.69007716	1.30669	0.000236501	-0.653021921	550.1452453	3.18929509	1.44105	2.0426E-06	1.70795817
474.8420083	0.67920036	1.30631	0.000212924	-1.194738006	550.1797368	5.74253188	1.36657	2.00892E-05	-0.782103108
307.9161	0.70452061	1.30619	0.000221693	-0.89883171	551.2728437	4.81185433	1.03581	0.005745661	0.597949679
459.2221853	8.6682566	1.30568	0.00019131	-1.31833532	554.8189784	0.69374003	1.29347	0.000115924	-1.096171605
588.8210924	0.6769372	1.30513	0.000211834	-1.331341858	559.2021594	7.32969422	1.48272	4.27735E-07	1.580900193
972.9923427	7.71983106	1.30285	0.00020164	0.933079357	560.7814969	0.70627741	1.28043	0.000145808	-1.18613594
228.0500668	2.88790945	1.30172	0.000249599	0.495790807	561.2211321	8.99745271	1.56797	1.14902E-09	2.789783181
229.1512942	1.0403623	1.30149	0.000247707	0.444081804	562.1991842	8.33613397	1.49574	1.92148E-07	-1.315409742
529.2184512	7.40832989	1.3013	0.000262737	-1.53068751	562.2156817	7.98407523	1.43716	2.25799E-06	-0.921125309
503.2030546	6.21225225	1.30083	0.000231542	-1.511061925	563.1430786	7.21544127	1.5423	1.26407E-08	-1.609585766
177.9628643	0.71365576	1.30061	0.000226144	-0.503162561	564.3328491	11.8716243	1.13551	0.00198657	0.911623595
252.0865769	6.25926113	1.30024	0.00024394	0.817876775	564.8306151	0.66819347	1.25064	0.000279467	-1.279472756
167.1062521	6.87693916	1.30023	0.000256338	0.737639221	565.1953645	7.4849598	1.14312	0.00142467	-1.744785557
625.2131843	8.54944852	1.3001	0.000214728	-1.021735688	565.215868	5.79518542	1.47032	6.10895E-07	-0.778222079
396.1651173	3.54618986	1.2985	0.00024673	-1.734103641	567.1856921	6.36437306	1.56476	2.43524E-09	4.12581954
382.6836852	3.76623479	1.29841	0.00038854	1.219107442	567.2831282	10.9775389	1.22038	0.000485326	1.328737383
125.9857307	0.67979172	1.29681	0.000316112	-0.609957028	569.1576248	7.3926831	1.54393	9.25161E-09	-0.877240789
766.2782484	6.61481834	1.29647	0.000227546	0.934280045	569.2250531	8.39261845	1.24764	0.000348303	1.308174996
319.1473501	1.04969847	1.2949	0.000331487	1.375258989	569.2984144	11.0942207	1.10504	0.002454849	1.099273321
607.2013481	8.52009681	1.29223	0.000255985	1.442884792	570.2172404	9.70773902	1.54552	9.35204E-09	-2.750158564
543.2543331	4.00304746	1.29188	0.000251858	1.087932345	573.2215987	8.66023678	1.14318	0.001412121	-2.793162854
161.0622437	5.75174128	1.29124	0.000268283	-1.537694084	575.1430587	8.78265646	1.51763	5.55672E-08	2.036179215
652.2554301	5.34142982	1.28985	0.000264487	1.093973875	575.14344	8.3491932	1.54922	6.98232E-09	2.544820603
271.6301548	7.50344099	1.28955	0.000268637	-0.938801304	577.2110796	7.70529022	1.36441	2.27546E-05	0.988871939
130.5260944	0.72703138	1.28828	0.000323072	-0.748092452	578.1746877	6.19677175	1.09128	0.003443088	0.68562963
290.0756343	0.75496261	1.28653	0.00031956	-1.252187659	580.1916181	6.40226649	1.52606	3.40347E-08	1.398244556
770.845735	7.93577656	1.2853	0.000277346	-1.198843344	581.1674255	6.10825396	1.51516	6.34771E-08	-1.520452345
362.1964423	6.89808481	1.28509	0.000293629	-0.918916443	581.1963687	6.88686148	1.41567	4.77946E-06	-1.394763736
874.1393799	7.4241773	1.28405	0.000291785	0.989945901	583.1246219	1.33505965	1.47069	6.00315E-07	1.764339852
167.0116702	0.6794222	1.28119	0.000411391	-0.540937463	585.2184003	7.46012927	1.08428	0.003301705	0.620076736
223.1325899	7.18614759	1.28104	0.000313655	0.793679762	585.2935664	10.5054227	1.20615	0.000580578	1.162486939
442.2163384	1.04755352	1.27684	0.000426747	1.772681785	587.2577542	4.79908655	1.19921	0.000679835	1.135883671
309.0840461	0.9731832	1.2764	0.000339489	1.640715396	587.2582299	5.01279633	1.45667	1.0909E-06	1.112549835

113.0597071	5.99238699	1.27607	0.000341777	-0.989014832	591.1960633	6.10971084	1.55346	4.27302E-09	-2.023129336
156.0397051	0.69974988	1.27508	0.000406889	0.521506448	593.1321325	10.1461658	1.20263	0.000609708	1.801293027
344.8814209	0.67792388	1.27454	0.000436013	-0.988112544	593.1518318	5.94422388	1.57669	6.00906E-10	-0.684608132
547.3607297	14.0781396	1.27426	0.000354023	-0.145435962	593.8740656	5.94187401	1.54611	1.39623E-08	-0.734275824
200.0435136	0.73567509	1.27218	0.000380441	-0.848734086	599.1191889	7.32766116	1.5346	2.08468E-08	-1.558291947
659.1588492	5.30478606	1.27177	0.000441658	-0.830985296	601.1397954	0.90521516	1.32449	5.67388E-05	1.055073623
220.8777522	0.70770154	1.27166	0.000373056	-1.069880271	601.192863	5.79782862	1.53296	2.1793E-08	-1.062847205
248.0736358	4.4067241	1.27043	0.000354608	1.004260354	603.1944323	7.32328412	1.41908	4.75092E-06	0.934847374
505.1321044	7.80450632	1.26858	0.000462848	1.821458373	603.2285526	5.64111012	1.50197	1.37286E-07	1.603871284
105.4734971	0.68211115	1.26586	0.000444244	-0.984546403	605.1537232	8.24065455	1.61317	1.60941E-12	2.280234907
699.3559797	11.3044246	1.26531	0.000452086	1.523007054	607.1705905	8.0752602	1.32749	5.635E-05	1.781625464
795.3767686	5.99168313	1.26514	0.000422359	-1.581548737	607.173797	8.7180878	1.18332	0.00083013	1.52624027
284.0968287	1.04789027	1.26409	0.000394472	0.641393431	607.2112207	1.64889363	1.23501	0.000358133	1.109629961
557.1198874	1.12793622	1.26334	0.000417804	-0.542085127	608.1859053	6.10765256	1.52875	2.7005E-08	-1.497739456
722.8178259	8.03088797	1.26309	0.000414888	-1.445807304	609.1482251	7.39707643	1.3228	8.2762E-05	1.045461357
428.2069662	1.04411342	1.26231	0.000439222	2.133335784	609.9467967	8.15544748	1.05157	0.004536797	0.409164291
514.2136415	5.83549275	1.26229	0.000472565	2.204564973	610.200938	7.47885293	1.45324	1.23112E-06	-0.992109724
353.2685284	11.3045159	1.26192	0.000520304	1.338356718	611.14721	0.98228584	1.44004	2.0522E-06	1.368648163
549.1247028	8.05585562	1.26118	0.000650066	-0.466282558	611.1636726	4.94612682	1.09001	0.003179846	0.419503098
499.1225762	6.08891894	1.25798	0.00043518	0.690736704	611.2211676	5.79738255	1.4798	4.28144E-07	-0.883353953
957.2852203	7.42370406	1.25797	0.000444626	0.936031493	613.0226969	1.16525803	1.55203	4.7636E-09	-2.247261062
534.2183899	6.19707582	1.25653	0.0004593	0.719218592	613.2143653	6.80359295	1.05572	0.00451325	-1.185994122
233.1170927	7.05903518	1.25563	0.000484163	-0.822838083	613.2158154	7.25536842	1.04054	0.005251403	-1.135533226
461.1269424	5.1139004	1.25352	0.000507041	2.689389313	615.2648033	8.77573632	1.06413	0.004008591	-0.803908768
441.173564	7.96735805	1.25279	0.000824732	0.783728275	616.3265622	5.85201319	1.17667	0.000931903	2.589949475
445.0864504	0.7455717	1.25141	0.000567141	-1.241357765	617.2457474	8.40206686	1.21456	0.000490503	0.754479012
365.1206195	4.56351987	1.25103	0.000495982	-2.306370672	619.2630909	8.98663384	1.50307	1.27351E-07	3.196603378
518.1803169	4.17176889	1.25037	0.000593203	1.62558245	621.1478255	7.25382156	1.00809	0.014983479	1.160352485
124.0360098	1.04074115	1.24906	0.00101984	0.816871238	623.1639926	6.18499955	1.16341	0.001367164	0.720305831
540.8836068	0.673816	1.24881	0.00056722	1.067576356	623.1642392	6.38773826	1.42453	5.97244E-06	-0.798813392
426.1969723	8.47051296	1.24845	0.000535153	4.214763002	625.1798353	6.22639232	1.18608	0.00117134	-0.894530068
453.2062015	8.4606875	1.24815	0.000508792	-1.580892646	625.180557	5.78743028	1.12254	0.003240937	-0.380120288
785.2146661	6.43024062	1.24754	0.000513685	1.076539265	626.1385362	7.3296111	1.41651	5.80183E-06	-1.821724787
685.7972292	7.47257684	1.24695	0.000519036	4.513913239	627.1228795	5.10232969	1.17059	0.001001424	-0.796036467
751.8714903	7.93827965	1.24658	0.000528666	-1.276341238	627.2090851	7.05892155	1.25143	0.000263117	-0.678415979
615.2264455	7.06618087	1.24556	0.000579849	-0.670463357	628.2114517	5.79683578	1.48811	3.06094E-07	-1.117768894
519.2646918	7.81031122	1.24525	0.001095209	-0.661904283	629.1299005	7.09818205	1.54067	1.70085E-08	-1.76813097
450.8768614	0.67872371	1.24257	0.000659351	-1.058429294	630.8762871	0.66778874	1.24481	0.000355753	1.031937659

223.132566	5.75132824	1.24207	0.00065154	-0.486847897	633.2060802	7.90651558	1.42883	3.12226E-06	-0.73712781
290.1021023	4.58236766	1.24055	0.000662304	-0.782951533	633.2425626	8.34912611	1.41898	4.23904E-06	-1.120260781
208.0966997	5.38525639	1.24011	0.000605686	-0.76818132	635.254614	7.06167947	1.31928	6.61501E-05	1.078870479
628.2006754	6.20271225	1.23871	0.000774114	-1.552549782	637.1862975	0.89875821	1.54353	9.4607E-09	1.36752957
591.1825153	4.00669299	1.2356	0.001706875	-0.681263281	637.2370357	7.04973114	1.03839	0.0050359	-1.088622691
519.1690925	5.83349237	1.23423	0.000676382	1.846169389	647.2931006	8.82207718	1.11895	0.002405381	-0.944341063
402.1581755	4.45971537	1.23379	0.000638721	-0.856340963	649.1038608	5.09772641	1.39093	1.16188E-05	-1.187416543
771.2341596	7.14893263	1.23378	0.000672261	1.015193246	651.2162837	6.90932638	1.28015	0.000146831	-0.904918345
477.1008555	5.11105396	1.23295	0.000740662	2.840510481	652.2178747	6.96162779	1.2455	0.000308065	-1.422600067
724.260042	6.94295906	1.23226	0.0006521	1.926423341	653.1391963	7.21482901	1.46131	1.66494E-06	-1.855546679
282.2787093	14.2712786	1.23125	0.000660205	-0.929301259	653.1783579	7.97917803	1.45343	1.22591E-06	1.815132617
330.1490906	1.01522747	1.23098	0.000701707	0.757806862	654.2270584	7.0589364	1.19756	0.00069663	-0.637653023
1034.602169	7.72661753	1.23035	0.000674463	0.949236124	655.2297886	8.67470249	1.21705	0.000466322	0.836025597
704.1912107	6.22800417	1.22986	0.000940697	-0.548232437	655.2972781	8.06013351	1.06736	0.00466993	0.822256894
458.8576458	0.67404216	1.22839	0.001000702	-0.998304434	656.1481708	7.0845968	1.49108	2.91478E-07	-1.614594387
275.0761512	3.69357648	1.22783	0.000931206	-0.852646578	659.2219824	7.40807932	1.21434	0.000567216	1.885665206
309.1327085	6.13673132	1.22701	0.000715004	-0.51567753	662.8334054	0.66786138	1.12873	0.002150112	-0.708616692
471.3858101	0.67361864	1.22384	0.001012418	-1.165460085	666.2263153	5.64120235	1.4847	3.20433E-07	1.004256736
169.1219803	6.39247323	1.22155	0.000766254	-0.728133989	667.2616609	7.80907856	1.09074	0.002941167	-2.836156502
669.2728014	8.82015264	1.22089	0.000813713	-1.033631788	669.2056916	8.54827007	1.24595	0.00027644	-0.944418921
211.0963427	3.39312307	1.21987	0.000846602	0.870141355	673.2406905	8.67695989	1.39187	9.91364E-06	1.35859747
394.9760874	0.7416549	1.21854	0.000826296	0.476152422	678.8111618	0.66791875	1.16162	0.001152066	-1.020850911
286.091631	1.69843159	1.21432	0.001080427	0.770725548	679.195795	0.92771745	1.3381	4.32257E-05	1.801023526
185.9623865	0.70077	1.21183	0.000930645	-0.767528131	681.278032	8.38776758	1.17207	0.000921229	-2.571918247
526.2343822	8.79945046	1.20851	0.001365132	-2.340600285	683.2931459	8.26720274	1.11107	0.002236763	-3.348743184
353.1711407	1.3839189	1.20571	0.001098977	-0.798032081	685.1272546	3.4510223	1.05942	0.003971785	-0.973825561
370.17091	4.11160356	1.20227	0.001066637	1.998846402	687.2885984	5.95315154	1.15644	0.001317751	1.235091702
167.0466753	4.40595552	1.20156	0.001072067	1.173105081	688.1746476	6.21719039	1.14033	0.002748131	-0.536927094
787.2296605	5.88129038	1.19902	0.001053838	0.683713196	689.2306902	6.45451674	1.39406	1.04138E-05	0.987129209
108.45167	0.70690896	1.19867	0.001093666	-1.077207815	691.1203379	5.94970506	1.03659	0.005372181	-0.269759129
278.0750794	6.18945968	1.19827	0.00106671	0.693049765	695.2717082	8.0550772	1.052	0.005246015	0.650487781
303.1916625	0.84553901	1.19411	0.001296267	-0.439622116	697.3668126	11.4342679	1.25307	0.000291858	1.414580603
426.1970746	8.30840141	1.19387	0.001201322	4.072023537	698.214665	5.74742794	1.40803	6.13794E-06	-2.304467207
467.2022534	5.03645876	1.19284	0.001175181	-1.9009591	699.1438278	4.91751912	1.06257	0.00602989	-0.505340884
403.2186485	4.87595949	1.19262	0.001726778	-0.808517613	705.2634699	9.19484019	1.59193	9.22312E-11	3.73407549
443.1483615	7.12812968	1.19259	0.001404208	-1.36468895	707.1848086	7.73987469	1.17398	0.001504691	-0.711331772
624.2691962	7.80094372	1.19151	0.001201534	-0.860427003	709.1640368	6.4441063	1.24248	0.000340153	-0.447365862
382.9307085	0.68033964	1.19105	0.001316429	1.191378986	709.2719976	8.61381165	1.38588	1.22768E-05	1.235125314

297.0607991	5.10179749	1.19045	0.001444429	-0.742023598	709.3569489	7.04968687	1.36683	2.00987E-05	-1.763953585
335.1335809	4.11117794	1.19014	0.001315381	2.166624249	713.158942	4.88758623	1.39967	1.08241E-05	1.378417133
697.2843318	8.06352712	1.18979	0.001266125	0.841486738	713.2313627	5.11075665	1.19721	0.000631064	0.507288637
448.2542211	5.9967588	1.18911	0.001404254	-0.631262754	714.2627595	8.13367137	1.09596	0.002651003	-2.341164884
389.1433047	5.15871518	1.18806	0.00145282	-1.031928037	721.1243894	4.91613337	1.1227	0.002293819	-0.768241303
394.1644776	3.25723718	1.18767	0.001433865	2.425203159	721.3667479	11.3052646	1.26514	0.000227633	1.433592649
507.7775393	5.72658373	1.18711	0.001340987	-1.927486603	723.2731644	8.6251593	1.48238	3.50334E-07	2.777286013
187.1099675	6.12993064	1.18663	0.001265732	-0.541528022	723.3820807	11.7467947	1.36115	2.97445E-05	2.270243006
198.0526327	0.79266333	1.18579	0.001366866	-0.661687135	725.1955251	5.74337722	1.31819	9.41598E-05	-0.845883843
360.1476373	4.29817291	1.18548	0.001764105	-0.75547489	725.1961019	5.31444543	1.38515	1.332E-05	-1.388373585
377.0456585	0.93676569	1.18467	0.002123012	1.097635072	725.397551	12.2784408	1.43965	4.28784E-06	2.545419183
330.5842717	7.90716255	1.18419	0.002008444	-0.429001834	727.1771945	5.55994562	1.30278	9.96256E-05	1.167453676
535.1428199	5.83592486	1.1825	0.001545015	2.710840262	729.3354541	5.98281421	1.10462	0.002913707	-0.821470705
204.9039935	0.70617295	1.18225	0.001384074	-0.537527833	730.2575293	7.80250251	1.14857	0.001376886	-2.472845216
1055.498144	9.60265975	1.18171	0.00291922	1.63417839	733.2939904	8.97787486	1.51832	6.28967E-08	-1.755536394
512.8945646	0.67484165	1.18151	0.001360199	0.94356753	735.1401634	4.88232248	1.4613	8.96151E-07	1.377254669
431.1526284	8.47585868	1.18141	0.001426163	3.29960709	735.2213329	1.25298452	1.53278	2.04204E-08	1.24358996
392.1317138	5.38210215	1.18061	0.001638236	-1.342965397	737.2381453	0.95057026	1.00903	0.007005216	0.591924368
401.0867957	8.9279196	1.17977	0.001550254	1.17539226	737.2722859	8.4014559	1.17895	0.000864388	-1.591016054
531.2429354	7.70360224	1.17947	0.001389928	-0.349412979	737.287086	6.76523894	1.46648	7.26999E-07	1.484995856
221.1169295	5.11129527	1.1791	0.001495665	0.398896912	738.3567687	11.3052646	1.27295	0.00019651	1.480972508
193.0856298	7.15839084	1.17803	0.001973349	0.667600035	739.2110653	7.05822442	1.35467	3.17213E-05	1.191810114
337.9689828	0.67404897	1.17756	0.001725361	1.222341348	741.2611816	6.84173442	1.23831	0.000328228	-2.106510169
1051.382197	6.43281116	1.17651	0.001479037	-1.035492178	743.2092904	8.14504968	1.12104	0.001864404	0.778764174
237.0870358	1.4196596	1.17605	0.001532791	-0.609750353	743.3134816	8.62339721	1.18011	0.00090886	2.501339409
795.2315459	7.00632416	1.17585	0.002307845	0.623493836	744.856154	0.66757338	1.24107	0.000420602	2.011159258
484.203072	7.80142807	1.17497	0.001726505	1.871132677	749.8576761	7.9455194	1.1223	0.001838625	-1.07597596
871.2499034	7.42360502	1.17149	0.001874599	-0.558044107	751.195308	5.11774141	1.18102	0.000961795	3.484390508
209.1452435	7.81769842	1.16911	0.001685705	-1.066501506	755.2059987	6.54412167	1.56332	1.8038E-09	2.103782499
500.1699472	6.88432352	1.16884	0.001628518	-1.078716405	759.2354717	6.23117542	1.0074	0.007013877	-0.592205956
462.2332966	4.60257797	1.16786	0.001657927	-0.5405315	761.1717327	5.74670125	1.3932	1.60938E-05	-0.820149388
267.6375128	4.10559547	1.16523	0.001699864	1.620125794	763.1873805	8.04010237	1.24999	0.000303117	-0.504257471
362.0937091	0.93175898	1.16232	0.002013735	-1.137430134	765.1924118	6.88076993	1.0347	0.00655452	0.888015283
258.1439436	1.5259923	1.16229	0.001843097	0.676074956	766.2270956	0.86368005	1.46697	8.69173E-07	2.921708072
507.8690604	0.67412309	1.16214	0.002031989	-0.876254743	766.250159	6.67649837	1.10908	0.002223354	0.938104543
401.1421514	8.70912255	1.16053	0.003433693	2.006274338	768.2039478	6.62951457	1.41825	4.5796E-06	1.350128171
208.0967522	6.48064165	1.15964	0.002454558	0.483878969	769.2395081	7.15035739	1.35498	3.14675E-05	1.257886084
130.0495999	1.20658961	1.1593	0.001790757	0.732913304	771.1645224	4.93641073	1.00317	0.007336808	-1.137981865

253.1795049	7.64191189	1.15831	0.001931483	-0.940184626	771.1646451	4.50148761	1.10434	0.003894566	0.636393841
364.702925	9.20529883	1.15709	0.002313974	-0.76933267	771.1648521	4.69893862	1.19387	0.000893268	-1.137547793
404.1527466	3.29279675	1.15708	0.005059759	0.435579882	771.2012016	5.16789866	1.50888	8.98546E-08	1.191186151
406.4033301	0.67428195	1.15699	0.002437849	-0.97516321	776.2724979	7.98288149	1.06938	0.003590759	-1.059509645
513.230512	7.69802913	1.15519	0.001891884	-0.456286512	776.3506309	7.74785913	1.48503	3.868E-07	-1.991947193
555.1487923	6.19201262	1.15413	0.002007061	0.480060385	777.2832928	7.50927753	1.05612	0.004361753	0.646495619
255.1223395	5.14498999	1.15399	0.001949309	0.31619021	779.2478706	1.84428971	1.51838	5.49765E-08	2.085080061
386.1092385	6.66566219	1.15365	0.001937841	-0.850284834	780.2508866	1.59433521	1.53496	1.75908E-08	2.909627766
546.2127293	5.16041512	1.15308	0.001939645	-1.441595587	783.2339444	6.43013816	1.1871	0.000778067	0.832344577
255.1932415	7.04024905	1.15304	0.001962014	-0.848510465	783.2363878	6.99465877	1.02164	0.006752034	0.596229514
414.1783242	3.55049002	1.15216	0.001960959	1.202296791	785.1798944	5.50248579	1.10635	0.003759783	0.768281146
548.2340065	6.05006266	1.15121	0.001983148	-0.494007477	785.2167882	5.87815175	1.16859	0.001134129	0.791514101
917.1130315	8.2951295	1.1501	0.002244316	-0.901957298	793.2259935	6.13005433	1.4109	6.44252E-06	0.872534667
503.1912423	7.4867181	1.14867	0.002106783	-2.01787679	795.2002191	7.04366244	1.57064	8.63345E-10	2.613779025
481.2181541	5.15239562	1.14839	0.002298565	-1.066203877	796.2187773	4.28592638	1.09033	0.003324639	0.649365594
143.1064206	8.31319932	1.14796	0.002312182	4.344457361	799.19547	5.56082751	1.2515	0.000295778	1.644312621
529.2396645	4.03023015	1.14736	0.003864888	-0.688892501	802.2078319	7.06060133	1.30241	0.000103309	1.022186565
220.1179231	2.99776011	1.1473	0.002102629	0.405696116	805.2011303	7.14196256	1.47791	4.95804E-07	0.983750372
572.8392628	0.6736878	1.14578	0.003234103	-0.839597296	805.3243135	9.02663785	1.06717	0.003951059	0.597446557
362.1973592	5.21755732	1.1457	0.002235243	-1.001239846	807.1768656	5.16789866	1.44748	1.5374E-06	0.873794142
502.2321975	8.04413663	1.14508	0.002245884	-1.12721097	807.3306517	6.7334999	1.43683	2.8654E-06	-1.531258985
182.9627049	0.68128596	1.14334	0.002223807	0.380875287	808.7689482	0.66832753	1.21691	0.000492016	-1.380130103
533.1543007	0.92843453	1.14179	0.002343499	-2.422846202	811.3036865	7.58372567	1.07584	0.003264196	0.451947959
463.0663045	5.52960439	1.14106	0.002552031	3.670826667	821.1921192	5.87798788	1.16824	0.001028995	0.6180118
609.1816815	8.07188836	1.13948	0.002327697	1.188589746	823.2164589	4.98982201	1.16522	0.001045016	4.657675825
919.9834372	6.43443922	1.13921	0.002385107	-0.95194566	829.4227563	9.1285928	1.19222	0.000686189	0.674090892
908.1062865	7.99119173	1.13853	0.002562278	-0.822363608	831.2444807	5.99789087	1.38024	1.39479E-05	0.987913095
459.1290251	5.86929946	1.1384	0.002402673	0.353698542	832.216307	7.14311409	1.467	8.19307E-07	0.996682137
526.7911188	6.6059695	1.13772	0.002390525	0.998366439	833.2747864	7.3055782	1.52896	2.76252E-08	2.313385268
478.1385633	0.98520429	1.13738	0.002358259	0.977195712	834.1951789	5.17354222	1.5142	6.70607E-08	1.056150525
689.2622237	4.98011527	1.13738	0.003387529	-0.537223706	835.2091905	0.96263881	1.09193	0.002734104	0.64451292
439.1236595	6.58409177	1.13692	0.002420346	-0.980508719	835.2893645	6.15687708	1.17687	0.001181495	0.6857836
456.1714426	5.11891424	1.13682	0.003026437	3.798062465	835.3611401	7.94349157	1.33024	5.32221E-05	2.805838846
750.3797202	9.20529883	1.13625	0.003598408	-0.445470354	841.350882	7.72595434	1.44819	1.66246E-06	-1.628397674
686.3024847	7.81040095	1.13622	0.002516718	-2.995621226	843.3294999	5.9861966	1.14413	0.001437369	-0.904200932
578.3295454	5.52974682	1.13566	0.002804687	3.537583924	845.2787457	0.83910373	1.39288	9.80272E-06	1.21055407
312.1654749	4.40345192	1.13489	0.002916454	1.731226503	845.3334064	6.71962178	1.54153	1.09741E-08	-1.225899966
440.148476	6.53035676	1.13253	0.00356845	1.956470536	847.2518514	7.01025858	1.03173	0.00603784	0.729565827

211.0962939	3.18238379	1.13242	0.002556754	0.937816147	848.2106909	5.87904735	1.07558	0.003494047	0.595378148
341.1380434	7.48465484	1.13105	0.002565549	-1.996466695	849.2581812	7.13394419	1.21071	0.000550182	1.929267784
296.9567621	0.67361864	1.13031	0.002590377	0.506086239	851.2986347	8.17082449	1.20059	0.000782277	2.861896186
384.1965614	1.04509033	1.12924	0.003291677	2.937357747	857.3664707	7.26439341	1.0156	0.007005236	-0.717768755
369.926017	0.67568619	1.12858	0.004465567	-0.596443671	860.231544	6.46006378	1.4344	2.52603E-06	1.26210663
317.1207866	4.40691793	1.12809	0.003013011	1.579801483	866.2580285	7.0868367	1.30725	8.58466E-05	1.546613168
446.187282	0.84005566	1.12666	0.003344866	1.088332346	869.2364662	5.86782643	1.38293	2.00376E-05	-0.707319005
242.0810505	4.60460056	1.12618	0.002753186	-0.548224232	874.8110437	0.66732764	1.36015	2.4067E-05	1.159581741
468.6893099	8.40273938	1.12593	0.003795456	0.60695471	877.3347108	9.15071026	1.13671	0.00175944	-1.051683173
289.0889641	1.66518102	1.12499	0.004260655	0.774069724	883.1844179	5.87089871	1.42887	3.10402E-06	0.868843912
520.3399673	11.8705113	1.12414	0.005480324	0.825928581	883.418682	10.8843346	1.38589	1.2841E-05	2.242060951
317.1207189	4.81191284	1.12165	0.003265428	-0.334755322	887.3335097	5.81506307	1.36575	2.17491E-05	-1.394166008
193.0857198	6.19201262	1.12133	0.002950905	0.499834154	887.3920703	4.6005016	1.41767	4.58903E-06	-1.194232901
398.1069002	8.9008674	1.12076	0.003941596	-0.624111255	887.4277614	7.26243471	1.40447	6.90459E-06	-2.244638723
105.0335585	5.10074982	1.12067	0.003596004	-0.795312701	889.3364274	6.11730472	1.54654	8.52235E-09	-2.190057569
816.4725641	6.1502287	1.11943	0.002916705	-0.999764181	896.409809	8.40407979	1.40662	6.32251E-06	0.894827243
428.1924904	4.51367452	1.11872	0.002993718	-0.586970102	903.2983687	7.42759105	1.46128	9.37227E-07	-2.056798346
384.1151228	3.22989499	1.11869	0.003404465	0.916734897	905.2941623	4.97103046	1.42984	3.96317E-06	1.595587115
746.9160555	6.74607415	1.11852	0.003028689	-1.591705257	909.3727775	4.59806679	1.2782	0.000155979	-0.943158154
668.193146	7.23823706	1.11803	0.004710106	1.528922929	911.3924167	8.59698783	1.14598	0.001382003	-1.744143718
382.1968867	1.05470929	1.1179	0.004331398	1.974412372	913.2669517	7.75354802	1.08597	0.005324453	1.400656087
374.1575814	7.76080807	1.11783	0.003592229	-0.597789935	919.252842	7.31354094	1.32786	5.2965E-05	-0.972001721
767.2038917	6.88421018	1.11744	0.003313341	1.056429685	927.2468461	1.23737451	1.51739	6.22825E-08	-1.952383493
329.1480576	5.61121733	1.1171	0.003497067	-0.301368778	927.2477073	1.0287884	1.19513	0.000654442	-0.814641644
370.0932508	4.92326519	1.11701	0.003551415	-0.690668004	931.2518189	7.31746832	1.42853	3.46966E-06	1.670155414
439.1443618	5.1151073	1.11695	0.003856623	3.200009813	931.2729062	5.05184732	1.48924	2.61197E-07	2.668809339
113.0344019	1.20658961	1.1165	0.003036792	0.500260066	932.3861266	8.40177164	1.33698	4.3485E-05	1.098964608
223.989149	0.68185646	1.11574	0.003077935	0.363674644	933.359697	7.0370892	1.1129	0.002371931	-0.802541214
249.0449	0.73257742	1.11343	0.003975814	0.19819778	937.3350488	8.11934301	1.02082	0.007641693	0.601876865
701.2262996	5.59220181	1.11318	0.00408223	0.341394938	943.3822993	7.38794069	1.55989	2.73295E-09	-2.132512729
305.0541418	1.04755352	1.11314	0.004023053	0.788695289	946.4108982	7.05165671	1.35364	2.84605E-05	-1.667861302
376.1758965	8.19559217	1.11309	0.003495686	0.607864221	947.2478255	8.02887919	1.42638	3.33591E-06	1.854693178
741.2234286	6.24640762	1.11149	0.003479918	-0.721263102	948.3340122	7.06001383	1.55938	2.82341E-09	-2.417330158
165.0543641	5.13606959	1.11017	0.0041255	0.838682071	951.2782169	6.73179378	1.33342	6.50278E-05	-1.096029943
736.2156998	6.43245869	1.10826	0.003468782	-0.928007503	955.3272459	7.63148885	1.34837	3.7037E-05	4.669363012
426.9024608	0.6736446	1.10715	0.004354076	0.407492165	959.3926936	8.40535702	1.08344	0.003220214	3.171892427
113.5317702	0.67066714	1.10652	0.003487725	0.318418418	961.2632401	8.21569583	1.34275	4.54769E-05	1.755868398
324.1265965	1.04490866	1.10609	0.004860251	-1.04733728	961.2633423	7.23319927	1.37604	1.91108E-05	1.638427444

705.2880829	5.63527343	1.10604	0.003807964	0.58468858	965.3633021	7.38858147	1.47626	4.9893E-07	-1.769376006
551.2003054	8.38729879	1.10538	0.003447153	-0.823273106	969.2578085	1.05562042	1.04317	0.005448839	2.175344821
580.295683	8.03641759	1.10401	0.005225364	1.573850489	969.3912644	7.99625485	1.04244	0.004901259	-1.138166558
935.3880687	8.5921446	1.10346	0.003681496	-1.046308096	971.2740252	1.05323552	1.41841	5.18924E-06	1.851042354
319.0813502	6.21354048	1.10097	0.006161569	-0.515603996	971.4858353	9.70055969	1.31617	7.59045E-05	-4.977776956
328.9001436	0.67404216	1.10093	0.005786726	-0.546319775	973.3472992	7.87344737	1.29169	0.000129883	1.888852748
400.9369134	0.72471142	1.09928	0.004364554	-0.653726622	974.4293519	7.98179078	1.28614	0.000140996	-2.086760192
765.8885314	8.12579652	1.09858	0.00371002	-1.101287129	977.2816301	7.99173478	1.48397	3.28402E-07	1.732476467
1011.508691	9.39115341	1.09816	0.00730274	1.029839643	979.130981	5.1013078	1.4166	4.94161E-06	-2.01323765
298.9896161	0.68936603	1.09701	0.00472771	0.44367878	985.3917539	7.55049918	1.29283	0.000116225	-1.677066684
482.1016368	0.9600902	1.09604	0.004621153	-1.182902857	987.3144759	7.73613682	1.0185	0.01180664	-0.475290144
245.0765138	1.21008201	1.09603	0.003882454	0.528071008	992.3037077	6.25591195	1.26455	0.000234802	0.841434651
458.1123476	1.14786578	1.09521	0.003885036	-1.490753002	993.2517725	5.97633019	1.12169	0.002053029	0.597254214
265.0176093	0.68339854	1.09495	0.003855659	0.231502017	995.3405199	7.8770833	1.07722	0.00323888	1.044281012
1018.005126	7.81109548	1.09447	0.004345764	0.945508247	997.3564131	7.93279343	1.09395	0.00407165	1.290287574
289.0882037	1.04054906	1.09327	0.004322567	-1.530703977	999.441364	7.97928063	1.40594	6.45871E-06	-2.28934875
358.2697844	7.99420664	1.09324	0.005130705	-0.731839471	1001.295006	8.33342826	1.06356	0.004091414	-0.761147324
557.2345491	4.03005458	1.09144	0.004330484	1.708660025	1003.328737	5.63262164	1.28153	0.000178313	1.647643342
744.2426161	6.24754176	1.09137	0.00423812	-0.744665797	1013.350684	7.99629524	1.03051	0.0073201	1.641607917
402.1561039	1.04035957	1.09135	0.00564616	1.397241471	1017.390885	8.4722412	1.15498	0.001321227	0.795040733
245.1857029	5.88518169	1.09046	0.005120863	-1.26882617	1023.369986	8.33671292	1.22306	0.000412158	-0.97773247
359.2280612	4.52282533	1.09006	0.004085304	-0.885424678	1035.334975	7.36897123	1.58776	1.58784E-10	-2.458471258
907.4347589	7.98989146	1.08981	0.005119021	-0.71442944	1038.020376	1.40851723	1.38542	1.79441E-05	1.929569418
538.1967658	6.55774059	1.08945	0.00472744	1.590470537	1039.294392	7.65782696	1.44168	2.5769E-06	3.068977616
282.0846691	0.76139193	1.08889	0.004275302	-0.585253948	1041.345879	7.94751508	1.28633	0.000155103	1.815130986
528.2736016	9.5462377	1.08814	0.004578672	-0.610318007	1049.378804	8.87553904	1.23673	0.00032544	-1.106236375
472.3103523	7.6012706	1.08795	0.00430452	-1.030135885	1053.401149	5.77967145	1.49797	1.646E-07	-1.581627824
912.4416493	8.01592808	1.08575	0.004273149	-0.792660546	1054.316959	6.23454919	1.29758	0.000102235	-0.529154139
817.9890539	6.43273434	1.08494	0.004518606	-0.924194589	1061.45806	7.55044326	1.22281	0.000417609	-1.585990836
610.2708112	7.06328052	1.08488	0.004551294	-0.754483908	1063.473895	7.61118337	1.30452	9.12085E-05	-1.181991214
310.0715436	7.90716255	1.08221	0.007348075	-0.371846724	1065.347244	7.59899463	1.40109	8.06215E-06	-1.34958535
600.1617734	7.90083133	1.08157	0.007867137	-0.364955913	1067.36113	7.37212466	1.07895	0.003217246	-0.659144322
333.094694	4.41080398	1.08139	0.005535387	1.826082367	1077.417782	8.89379084	1.28447	0.000151246	-1.095127527
493.1933903	5.33637024	1.08021	0.005159192	1.027392584	1079.397006	7.95552412	1.31462	8.63913E-05	-1.139202567
502.2639715	5.99180491	1.0795	0.004616663	-0.739678754	1085.262403	5.14916677	1.42586	3.85156E-06	0.771696564
651.1165738	3.64909994	1.07929	0.006289175	-0.830635786	1087.332307	8.34233961	1.19518	0.000661237	1.301531566
326.1291283	4.5862622	1.07906	0.005875922	-0.577142545	1091.362002	7.68525935	1.25296	0.000375972	-0.661189589
983.3328582	7.38026531	1.07771	0.004674285	-0.391166506	1099.422229	7.88615621	1.18216	0.000841006	-1.173023988

334.8679708	0.70094867	1.07706	0.006186935	-0.608566879
458.2755772	5.99548947	1.07227	0.004932802	-1.055385516
369.0820446	7.67706315	1.07063	0.013728511	-0.217879664
521.2019126	5.83201367	1.06972	0.00517857	-1.768898674
295.1361046	4.81110343	1.0674	0.007474813	0.558385116
274.9461126	0.67255041	1.06696	0.005670336	-0.905067808
635.4130683	14.0362125	1.06484	0.006025976	-0.182185389
495.1867164	6.05336337	1.06479	0.005432611	-0.425121404
312.1291822	2.90214722	1.06413	0.005925851	2.672589883
115.0753597	4.40469413	1.06226	0.006861196	1.956305628
351.1661843	4.84012224	1.0595	0.008181207	0.647865399
981.9918332	8.76091646	1.05917	0.005689307	-1.355170391
696.2760895	5.57968765	1.05895	0.005689708	0.615649587
539.1740068	6.19374491	1.05889	0.005682127	0.434559534
435.1290148	7.89852175	1.05845	0.008357204	-0.553841549
412.1466961	4.28442463	1.05819	0.005722184	0.471763751
245.0723205	1.03264987	1.05611	0.00843515	-0.740484096
294.1045824	6.00245182	1.05561	0.007525472	-0.528244649
728.3980229	9.20428681	1.05545	0.007452376	-0.523714781
627.1929597	6.23178529	1.0528	0.006352479	-0.781698038
328.0999435	8.05187801	1.05203	0.006318038	-0.478068159
177.0544172	7.67812605	1.05189	0.014421409	-0.203893889
434.1913953	1.04089562	1.05133	0.01063683	1.910936087
293.1233289	8.14163177	1.04895	0.010264343	-0.386931796
337.5922333	8.89335069	1.0484	0.010247791	-0.588193551
448.1691557	1.04863058	1.04834	0.009473702	0.993827811
602.5824768	6.72107468	1.04742	0.007906193	1.197874864
473.1077568	8.7045124	1.04712	0.006771842	-0.497894351
158.1537063	10.6332997	1.04676	0.010155495	-0.188028321
714.2069532	5.74149455	1.04636	0.006455919	-1.496708085
639.226529	8.05585562	1.04606	0.006475701	-0.426083222
191.0725558	6.80366155	1.0425	0.007376443	-1.380883982
436.217743	8.64398713	1.04155	0.010416992	1.524534329
505.2435661	8.27028811	1.04122	0.00739098	-3.804401459
359.1310796	7.80790061	1.03846	0.007096242	1.012446452
471.2610381	0.99618157	1.03629	0.007414112	1.095779379
398.1661822	6.4611819	1.03547	0.007656247	1.791462747
317.0802157	8.89417117	1.03473	0.012315132	-0.55523207

477.1739178	6.05259896	1.03465	0.007292247	-0.627365321
319.2034591	5.69424998	1.03449	0.010431285	-2.044992419
445.2222612	7.81171944	1.03341	0.007892473	-2.857803012
353.1684611	1.04408577	1.03287	0.007773992	-0.733823887
936.5055496	7.88412193	1.03227	0.007836627	-1.027858351
513.1968023	6.05251587	1.03186	0.007480048	-0.405247156
665.1716691	7.23751415	1.03149	0.010200035	1.295128959
368.1913118	8.71083008	1.03009	0.007668623	-1.546035142
511.201101	6.87695631	1.02873	0.007710473	1.064294477
403.1222819	6.46309548	1.02867	0.007915669	1.628872044
515.1241722	7.22227211	1.02798	0.016528667	1.42618817
410.926097	0.6794222	1.02763	0.008289381	0.645180414
300.2012325	4.71677626	1.02667	0.008201219	-0.876755048
250.1774626	10.4536869	1.02495	0.010160973	-0.414175859
766.3534768	9.2053345	1.02462	0.01686318	-0.312013948
321.560525	7.67812605	1.02357	0.009905701	-0.379177812
266.1038932	0.76478647	1.02315	0.008167331	-0.880884017
707.8694226	6.86859025	1.0228	0.012885481	0.905575933
198.9400965	0.67798697	1.02264	0.014396633	-0.312601443
525.2304152	8.99981643	1.01952	0.008636449	-0.852112948
511.2288918	4.19344589	1.01806	0.008647638	0.834851659
305.0631401	1.66713528	1.0168	0.011136325	0.718587468
129.0234643	0.66639494	1.01464	0.010347524	-0.573146335
483.1630914	4.33865307	1.01409	0.008903482	-0.515672639
727.2444465	7.89631143	1.01179	0.013053326	-0.627885808
401.167948	7.55663393	1.01174	0.010322811	-0.650171452
333.1908761	8.15889178	1.0113	0.00921702	1.387378864
419.13435	7.89922525	1.01037	0.014367814	-0.43321986
465.139671	6.21099053	1.0099	0.01403079	-0.54704809
447.1348001	6.99283237	1.00975	0.009681774	1.273372304
423.1627189	9.31958034	1.00887	0.009908606	1.320105932
527.2386002	8.97332363	1.00883	0.014798805	1.280799186
257.0778048	4.40532483	1.00876	0.00931937	0.61416354
339.1075142	7.30262931	1.00745	0.011411161	0.640629215
284.1338418	1.66610922	1.00427	0.013609238	0.730870934
367.1868868	1.49893771	1.00408	0.010027017	-0.441992931
316.0935425	1.00004794	1.00377	0.011668262	0.58317337

Note: M/Z refers to mass-to-charge ratio.RT refers to retention time,min.VIP refers to the variable importance in the projection of PLS-DA. Fold change (M/W) refers to the ratio of 'HAL'mean of normalization m/z value to 'AL'mean of normalization m/z value.The significant difference of m/z was screened by the criteria of VIP>1 and p-value<0.01.

Table s2 The primers used for PCR confirmation of the genes involved in citrate synthesis, transport, and degradation or utilization

Gene Name	Description	Sequence (5'-3') Forward primer	Reverse primer	Product size, bp	Sequence ID
PEPC1	phosphoenolpyruvate carboxylase	TGTTGGAAGAGGAGGTGGTC	TTAACCGGTGTTCTGCAAGCCAGCA	951	orange1.1g002089m
PEPC2		ATGGCTGCTAGAAATTTGGAGAAG	GCTTTGCGACCTTTACAAGC	783	orange1.1g002112m
PEPC3		TCTTCTTTCGCCTCTATGCCACTG	TCAGCAGCAACACGGAAGGTAT	581	orange1.1g001537m
CS1	citrate synthase	GAGGCTTATGAGAAGGGAAT	CTGAGAGCAGATTCCAAGAC	756	orange1.1g012107m
CS2		ATGGCGCCAAGCGTAGAATCATCGT	TTAATTCCCTGATCCAGCGAGTCTT	1542	orange1.1g010304m
NAD-IDH1	NAD-isocitrate dehydrogenase	GATCCCTCCCTTTCCTGAAG	CATGGCTGATGAAAGAAGCA	917	orange1.1g018224
NAD-IDH2		TCTCAACCACAAACCCCAAC	GATGATCGCAGATTGCCTTT	1062	Ciclev10025889m
NAD-IDH3		CCCTACCGATCCTCAAACAA	GGCAGCAATAACAGCATCAA	1094	orange1.1g017413
NADP-IDH1	NADP-isocitrate dehydrogenase	ATCCCATCGTTGAAATGGAC	TGGAGGCTATGCTGTTTGTG	975	orange1.1g015012
NADP-IDH2		AGGTTACCGTAGAAAGCGCT	GCAGCCTCCAACTTCTGAAC	919	Ciclev10005058
NADP-IDH3		GATGAAGTGCGGGTTAAGGA	CCTTTCTGATGAACCCGGTA	797	orange1.1g009041
GS1	glutamine synthetase	ATGTCTCTTCTCAACGATCTCCTCAA	CTAAGGCTTCCAGAGAATGGTGGTT	1071	orange1.1g013478m
GS2		ATGTCTCTGCTCTCAGATCTCCTT	TCATGGCTTCCACAGAATAGTGGT	1071	orange1.1g018391m
GS3		ATGGCGCAGATTTTGGCACCTTCTA	CTTGGGCATGAACATTAACTTCG	1449	orange1.1g018434m
PEPCK1	phosphoenolpyruvate carboxykinase	CCAGAACACCGGATCAAAGT	CCAGCAACCAGAGCAGTGTA	794	orange1.1g005865m
PEPCK2		ATGGCAGCGAACGGTAACGGAGAGTT	TTAGAAATTTGGACCAGCTGCAAGG	1995	orange1.1g006486m
FBPase1	fructose-1,6-bisphosphatase	TCGGCTCTCTCCTTACCAAA	ATACATGGGATCCAGGGTGA	751	orange1.1g015111m
FBPase2		CCGTTGGATGGGTCTTCTAA	TGCTTGCCAGTGAAAGATTG	605	orange1.1g019437m
CsPH8	p-type proton pump	CCGTGAAGGAATTGATTTGG	GCCAATTGCCAGTGTAACAG	877	Shi et al.(2015)
VHA-af	VHA assembly factor	ATGGTATCCGAATCCGACAA	TCAAAGCTCGTGCCTACT	633	orange1.1g028366m
VHA-A	V-type ATPase A subunit	AACACACAAGCCTCTCTCGG	ATCCAGACCCCAGAAGACCT	1066	Ciclev10030969m
VHA-B	V-type ATPase B subunit	GCTGGTTTAGTCAAGCGATT	AAAGATGCGAAGAAGTGTCC	846	orange1.1g011329m
VHA-C	V-type ATPase C subunit	TACTGGGTGGTGTCTCTTCC	TTGGGGACAACAGCAAGAAG	557	Ciclev10015638m

VHA-D	V-type ATPase D subunit	GGTTCCCACAGTGACAATGC	TTCAAGCGAAAGAAATCCTC	585	Ciclev10009240m
VHA-E1	V-type ATPase E subunit	CGGTTTATCCGCCAAGAA	CCACCACGACACCTCCTG	535	orange1.1g027450m
VHA-E2		TGAGGTTCATTCGCCAGG	CAACTCCACCCGAGCAAG	531	Cs6g10330.1
VHA-F1	V-type ATPase F subunit	ACTGGATTTTTGCTGGCTGG	GGTCATACGGATGGTCCTTAGA	247	Ciclev10002833m
VHA-F2		ATGGCTAATAGACCTCAAATACG	TCAGCGCCTGTCAGATGC	468	Ciclev10022651m
VHA-G	V-type ATPase G subunit	ATGGCATCTAATAGGGGTC	TTAATTCTTCACAGTTGTAACA	333	Cs6g11650.2
VHA-H1	V-type ATPase H subunit	ATGCACAAAGATCCCCTATTC	TCAGGCCTGCAAAAAGCTCGT	312	orange1.1g034108m
VHA-H2		CAGTGGAGTACTTGGCAACTA	TCCTTCAAACCTTCTTCCAGTTG	250	Cs7g14520.1
VHA-a1	V-type ATPase a subunit	CAGGTAAAGCGATGCGGA	AGGCGATTCCATAGAGTCCA	963	orange1.1g003454m
VHA-a2		TCATCCCTATTGAATCCGCT	AAAATAACATAACGACCGCC	1338	Cs8g08330.1
VHA-c1	V-type ATPase c subunit	CACCGTTCTTCGGCTTCCT	TTATGTTTGCGTGCATGA	425	Cs8g07570.1
VHA-c2		ATGTCATCGACTTTCTCCGGC	TTATTCTGCCCGAGATTGGCCT	498	orange1.1g031149m
VHA-c3		GTATGGGACGGCGAAGAGTG	CAAGCGAGACCCGAAGACAA	213	Cs1g25080.1
VHA-c4		GTGGCGATGAAACCGCTC	ACCTGACACCAGCATCACC	351	Ciclev10002781m
VHA-c''	V-type ATPase c'' subunit	ATGTCGGGCTCCGTAATGTTG	TTACACTGGTTTTGCAGGCCAT	549	Cs4g20460.1
VHA-d	V-type ATPase d subunit	ACAATTTGTGCCAGTGCGA	TACGACGATCATCTCGGGTG	615	orange1.1g018709m
VHA-e	V-type ATPase e subunit	ATGGGGTTTTTGGTGACA	TCACTCCTCTTCACTCAG	213	Ciclev10010112m
VHP1	V-type Ppase	CAGTGGTTCATCGTCTCCCG	GCCTCAAAAAGTCCCTCCCA	593	Ciclev10024946m
VHP2		CAGCCCTGTCCAAGATGTCG	GGACCAAGAGTTCTGGCGTG	840	Ciclev10007524m
VHP3		TGCCGTTGCTGCTTATGC	TGGGTGAGGCATCAGTGG	436	orange1.1g040141m
VHP4		GTTTCGCAATACAACCCCTC	GCTGCTGGCTCATCTCTACA	1247	orange1.1g003697m

Table s3 The mass spectrometric parameters for the positive and negative ion modes

	Positive ion mode	Negative ion mode
Capillary voltage	4 kV	3.5 kV
Cone voltage	35 kV	5 0kV
Iron source temperature	100℃	100℃
Desolvation temperature	350 ℃	300 ℃
Reverse cone gas flow	50 L/h	50 L/h
Desolvation gas flow	600 L/h	700 L/h
Extraction cone voltage	4 V	4 V
